# Propagation of alpha-synuclein pathology: hypotheses, discoveries, and yet unresolved questions from experimental and human brain studies

**DOI:** 10.1007/s00401-015-1485-1

**Published:** 2015-10-07

**Authors:** Toshiki Uchihara, Benoit I. Giasson

**Affiliations:** Laboratory of Structural Neuropathology, Tokyo Metropolitan Institute of Medical Science, 2-1-6 Kamikitazawa, Setagaya-ku, Tokyo, 156-8506 Japan; Department of Neuroscience, Center for Translational Research in Neurodegenerative Disease, McKinght Brain Institute, University of Florida, 1275 Center Drive, PO Box 100159, Gainesville, FL 32610-0159 USA

**Keywords:** Focal Lewy body disease, Hyperbranching axons, Molecular template, Structural template, Gradient, Alpha-synuclein

## Abstract

Progressive aggregation of alpha-synuclein (αS) through formation of amorphous pale bodies to mature Lewy bodies or in neuronal processes as Lewy neurites may be the consequence of conformational protein changes and accumulations, which structurally represents “molecular template”. Focal initiation and subsequent spread along anatomically connected structures embody “structural template”. To investigate the hypothesis that both processes might be closely associated and involved in the progression of αS pathology, which can be observed in human brains, αS amyloidogenic precursors termed “seeds” were experimentally injected into the brain or peripheral nervous system of animals. Although these studies showed that αS amyloidogenic seeds can induce αS pathology, which can spread in the nervous system, the findings are still not unequivocal in demonstrating predominant transsynaptic or intraneuronal spreads either in anterograde or retrograde directions. Interpretation of some of these studies is further complicated by other concurrent aberrant processes including neuroimmune activation, injury responses and/or general perturbation of proteostasis. In human brain, αS deposition and neuronal degeneration are accentuated in distal axon/synapse. Hyperbranching of axons is an anatomical commonality of Lewy-prone systems, providing a structural basis for abundance in distal axons and synaptic terminals. This neuroanatomical feature also can contribute to such distal accentuation of vulnerability in neuronal demise and the formation of αS inclusion pathology. Although retrograde progression of αS aggregation in hyperbranching axons may be a consistent feature of Lewy pathology, the regional distribution and gradient of Lewy pathology are not necessarily compatible with a predictable pattern such as upward progression from lower brainstem to cerebral cortex. Furthermore, “focal Lewy body disease” with the specific isolated involvement of autonomic, olfactory or cardiac systems suggests that spread of αS pathology is not always consistent. In many instances, the regional variability of Lewy pathology in human brain cannot be explained by a unified hypothesis such as transsynaptic spread. Thus, the distribution of Lewy pathology in human brain may be better explained by variable combinations of independent focal Lewy pathology to generate “multifocal Lewy body disease” that could be coupled with selective but variable neuroanatomical spread of αS pathology. More flexible models are warranted to take into account the relative propensity to develop Lewy pathology in different Lewy-prone systems, even without interconnections, compatible with the expanding clinicopathological spectra of Lewy-related disorders. These revised models are useful to better understand the mechanisms underlying the variable progression of Lewy body diseases so that diagnostic and therapeutic strategies are improved.

**Electronic supplementary material:**

The online version of this article (doi:10.1007/s00401-015-1485-1) contains supplementary material, which is available to authorized users.

## Introduction

Eosinophilic inclusions with halo, now known as Lewy bodies, (LBs) were identified by Friedrich Heinrich Lewy in the dorsal motor nucleus of vagus (dmX) and substantia innominata in the brains of patients with Parkinson disease (PD) [43, 69]. Initially, their presence in the substantia nigra (SN) was not readily recognized probably because they were less frequent in SN and their relevance to PD had not been established until Konstantin Nikolaevich Tretiakoff identified a consistent association between nigral degeneration with LBs as a pathological substrate for PD [73, 114]. However, it remained unexplained how these inclusions at multiple brain regions were related to characteristic motor deficits of PD until dopaminergic deficit of nigrostriatal system was identified as a major mechanism of PD [85, 164], largely involved in the motor presentation of PD. This nigra-centered explanation was reinforced after dramatic improvement by replacement therapy with levodopa [6, 39].

Histochemical identification of Lewy pathology was improved by silver impregnations [175] and later by ubiquitin or neurofilament immunohistochemistry (IHC) [71]. Ultimately, IHC for alpha-synuclein (αS), which is its major molecular component [168], is the most sensitive and specific gold standard to detect various types of Lewy pathologies. Amorphous early αS deposits in the neuronal cytoplasm, known as pale body and pale neurites [92], suggests that initial amorphous αS deposits may evolve progressively into more aggregated typical LBs.

In addition to this progressive aggregation of αS in the neuronal cytoplasm and neurites, the identification of LBs in the cerebral cortices in patients with atypical dementia [102, 103, 141] and in peripheral tissues [180] raised attention for their more extensive distribution that might explain other related clinical manifestations. Even after the identification of αS as a major component of LBs, however, it remains to be clarified how (when and where) LBs are formed [22, 45]. Because Lewy pathology is more abundant in the lower brainstem in a majority of cases, it is generally assumed as if Lewy pathology is initiated in the lower brainstem and spreads into upper brainstem [21, 45]. This idea led to a hypothesis that conformational change of αS to form aggregates might have templated itself to spread along neuroanatomical connections, which may be similar to prion disease, at least partly. Because this unified hypothesis is highly convenient and attractive to explain both local molecular change of αS as “a molecular template” and its stereotyped spread as “a structural template”, this combination is now enjoying the initial honeymoon of a happy marriage, when everything is usually considered in favor of this hypothesis. To make this marriage profoundly fruitful or at least acceptably fruitful even after the honeymoon period, some skepticism may be useful to recognize its potential pitfalls, possible misunderstandings or misinterpretations, if any.

In this review, we will first discuss the molecular basis for some of the animal experimental studies that may support a conformational templating of αS protein, as a pivotal mechanism involved in neuroanatomical spread of αS pathology. However, we also consider how this simplistic mechanistic view does NOT fully take into account the global complexities of induced experimental paradigms and the variable presentations of human neuropathology. We explore the additional biological changes and unique neuroanatomical properties associated with Lewy pathology that might provide alternative explanations for its distribution and clinical presentations. Indeed, specific neuroanatomical and morphological properties of select neuronal populations combined with alterations in multiple synergistic pathogenic mechanisms likely explain the variable clinical presentation of neurodegenerative diseases associated with Lewy pathology. αS pathology can often present with concomitant cerebral Amyloid β-protein (Aβ), tau and/or TAR DNA-binding protein-43 (TDP-43) inclusion pathology which could influence both the formation of αS pathology and the clinical findings in some patients [86, 104], but these topics are beyond the scope of this review.

## Molecular template (experimental considerations)

### Self-templating of αS promoting in vitro amyloid aggregate formation

**Fig. 1:**
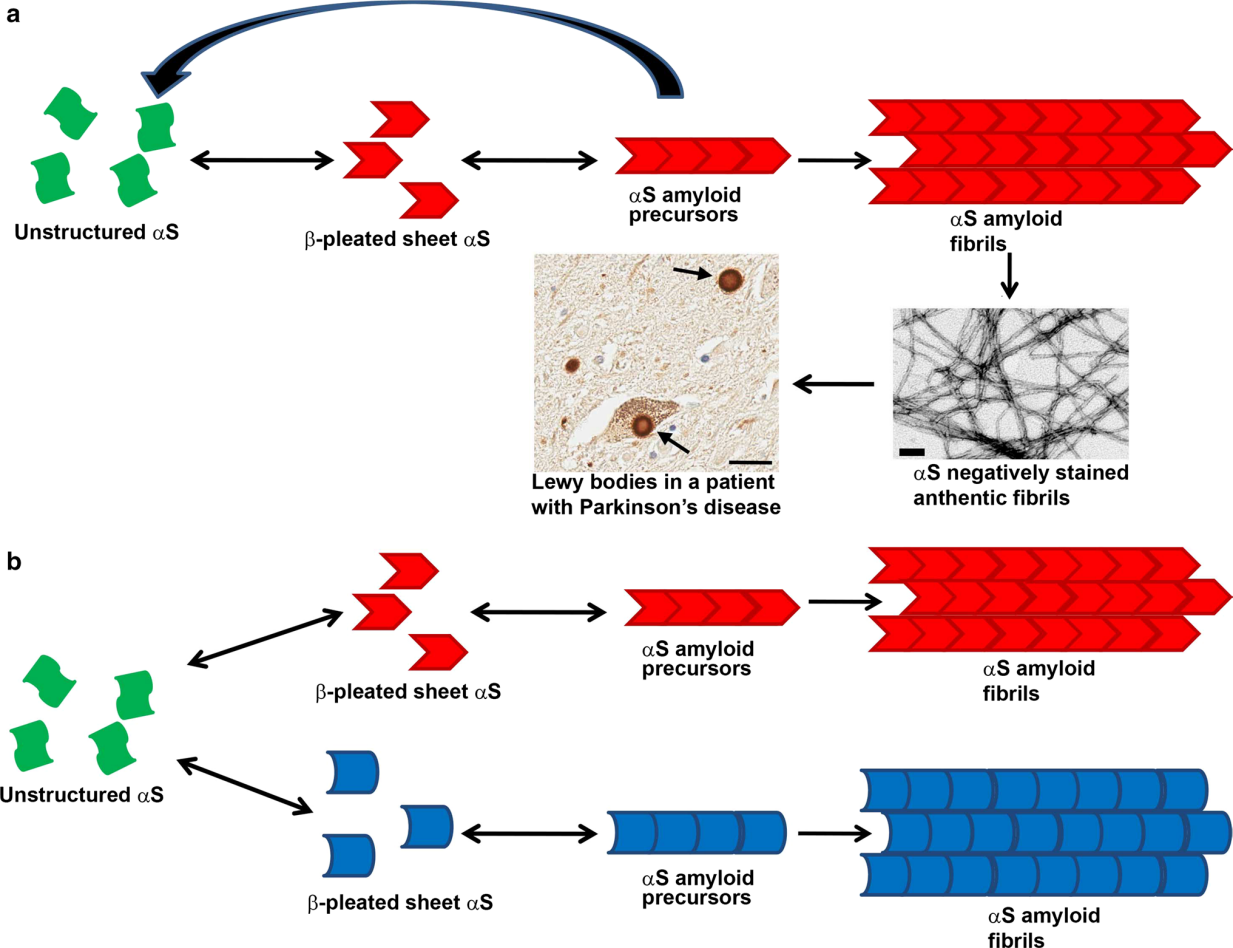
Schematic representation of the molecular changes resulting in αS pathological inclusions. **a** αS is naturally predominantly an unstructured, soluble protein (*green shapes*) that can randomly convert to acquire a β-pleated sheet conformation (*red shapes*). Once in this conformation αS can polymerize into longer amyloid precursor units and eventually fibrils (shown as negative stained αS fibrils assembled in vitro and observed by electron microscopy; *bar* 100 nm) that coalesce to form pathological inclusions (shown as Lewy bodies: LBs, staining with an anti-αS antibody; *bar* 25 μm). **b** αS can potentially polymerize into amyloid precursor units and amyloidogenic fibrils that at the molecular level have subtle conformational differences (*red shapes* versus *blue shapes*) and these are not compatible for co-polymerizing resulting in a “strain”-like specific polymers

The in vitro polymerization of soluble monomeric αS into fibrils that are biochemically, morphologically and structurally similar to those in human pathological inclusions is associated with a profound transformation in secondary structure from natively, unfolded random coil to predominantly β-pleated sheet [35, 36, 66, 79, 138, 166]. αS folded into β-pleated sheets is permissive to polymerizing into ~10-nm-wide unbranched fibrils that have typical amyloidogenic properties (Fig. 1) [36, 67, 68, 166, 186]. In vivo, αS is predominantly a neuronal soluble, unfolded protein [28, 60, 87], but a significant proportion is loosely associated with various cellular membranes driven by its ability to form amphipathic α-helical structures [42, 87, 98, 196]. Therefore, in its naïve state(s), αS is not primed to aggregate into amyloid-type inclusions.

The in vitro polymerization of soluble αS into amyloid fibrils is characterized by a lag phase followed by a rapid increase in fibril formation that can be promoted by increasing αS protein concentration [118, 138, 177, 191]. Therefore, it appears that the formation of a critical amount of αS amyloid precursors that may stochastically form in solution and is favored by increased protein concentration, which may also stabilize these structures due to increased protein interactions, is an important limiting step in driving αS amyloid formation. Experimentally, the simple addition of preformed fibrillar αS precursors (PFSPs), also termed “seeding” or “nucleation”, to overcome this threshold can rapidly promote the recruitment of unstructured αS molecules into amyloidogenic permissive structure resulting into a synergistic formation of amyloid fibrils (Fig. 1) [191]. This in vitro process of using PFSPs to convert normal αS into amyloid precipitating the formation of mature pathological inclusions has been termed “conformational templating” and provides an interesting model that could explain, at least in part, the spread of αS pathology that tracks with progression of neurodegenerative process in human brains.

### αS mutants accelerating or decelerating aggregation

Six missense mutations in the αS gene, *SNCA*, resulting in six different amino acid substitutions (A30P, E46K, H50Q, G51D, A53T, and A53E) have been identified that can cause PD with or without additional clinical features such as dementia reflecting a more widespread presentation of αS inclusion pathology typical of dementia with LBs (DLB) [5, 96, 107, 116, 145, 150, 151, 194]. In addition, short chromosomal duplications or trisomies containing the *SCNA* gene, plus relatively short flanking regions on chromosome 4, can also result in PD or DLB [31, 56, 167], indicating that a 50 % increase in the expression of αS is sufficient to cause these disorders. Genetic alternations resulting in increased αS expression are consistent with the notion described above that a higher abundance of αS can lead to a greater stochastic formation of αS PFSPs followed by conformational templating resulting in the spread of αS pathology. Alternatively, increased αS expression could also increase the focal formation of αS pathology with distribution perhaps reflecting selective vulnerability.

Consistent with conformational templating playing an important role in the spread of αS pathology, the A53T, H50Q and E46K αS mutations have consistently been shown to increase the rate of self-aggregation by these mutants [33, 35, 61, 67, 72, 95, 118, 138, 158]. While some reports suggest that A30P αS forms amyloid fibrils more slowly than wild-type (WT) αS [37], this finding has not been consistently observed by others [67, 138]. In addition, the A30P mutation appears to affect αS independently of protein aggregation, probably by partially impairing the ability of αS to bind to brain vesicles, likely due to a decreased likelihood to form α-helices [30, 52]. However, both the A53E and G51D αS mutations have been shown to significantly reduce the intrinsic propensity of αS to polymerize and aggregate, hence in principle, lowering amyloid transmissibility [55, 62, 157, 158], despite still causing early-onset PD with robust αS pathology [96, 116, 145]. Collectively, there is no simple explanation for how all the known missense mutations in αS result in PD and their divergent effects on in vitro aggregate formation suggest that conformational templating cannot solely account for disease progression.

### Cellular models of αS release, uptake, transfer and inclusion formation

Many cell culture studies have demonstrated that several non-mutually exclusive mechanisms including release by exocytosis [109, 121], uptake by various endocytosis mechanisms [1, 110, 112, 171], release/uptake of exosomes [41, 53], and release due to cell death could be involved in the intercellular transmission of αS aggregates. Using transfection reagents to drive membrane permeability, it was shown that the entry of a small amount of exogenous PFSPs into cells expressing αS is sufficient to efficiently induce endogenously expressed αS to form mature cytoplasmic amyloidogenic inclusions [126, 140, 188]. Thereafter, it was shown that the simple addition of extracellular PFSPs to primary neurons may be able to induce the formation of intracellular αS inclusions without the addition of other reagents [179], with the caveat that these studies were predominantly documented by immunostaining with antibody pSer129/81A (see below). Utilizing microfluidic devices to separate the soma and axonal projections from separate but adjacent and synapsed primary neuronal populations, it was shown that following neuronal uptake, PFSPs can move by both anterograde and retrograde axonal transport but that synaptic contact is not required for transfer between neurons [58, 179].

Using recombinant adeno-associated viral (rAAV)-mediated αS overexpression in mouse primary neuronal–glial culture, it was demonstrated that exposure to exogenous PFSPs could efficiently induce intracellular αS inclusion pathology in cells overexpressing αS, and that this inclusion formation occurred in a fashion that was consistent with conformational templating since the morphology of the induced αS inclusions could be dictated by the type of PFSPs added [163]. Collectively, these cell culture studies have shown that under defined conditions conformational templating of αS leading to inclusion formation can occur and they have provided insights into the cellular mechanisms that might be involved. However, it should also be considered that the concentrations of PFSPs used for most of these studies are beyond the physiological levels of exogenous αS.

### Anti-phosphorylated Ser129 αS antibodies can label other phospho-proteins and αS phosphorylation is induced by stress

Although phosphorylation of αS at Ser129 can be a robust marker of αS inclusion pathology (Suppl. Figure 1) as this residue is highly phosphorylated in human pathological inclusions (~90 % phosphorylated in inclusions versus ~4 % phosphorylated in total brain αS) [3, 24, 59, 187], phosphorylated Ser 129 (pSer129) immunoreactivity on histological sections should not be equated to the formation of αS inclusion without validation with other techniques. First, most pSer129 αS antibodies cross-react with other phosphorylated proteins. For example, αS pSer 129/81A antibody that has been used in many studies can strongly cross-react with phosphorylated low-molecule mass neurofilament subunits (NFL) and can give the false impression of αS inclusion pathology (Suppl. Figure 2) [162]. Not all αS pSer 129 antibodies that have been used in other studies also cross-react with phosphorylated NFL, but antibodies such as MJF-R13(8–8) can cross-react with other non-αS phosphorylated proteins (Suppl. Figures 1, 2). These facts highlight the need for well-characterized pSer129 αS antibodies (e.g. as shown in Suppl. Figures 1, 2), especially for immunostaining studies. It is not surprising that antibodies to phosphorylated αS Ser129 can cross-react with other phospho-proteins since this epitope can be phosphorylated by multiple kinases, such as casein kinases and polo-like kinases [24], and thus this phospho-epitope has a high level of homology to many phosphorylation sites in other proteins that are also recognized and phosphorylated by these kinases. Second, many cellular stresses or insults can greatly increase the phosphorylation state of many proteins, including αS Ser129, without inducing inclusion formation [187]. Therefore, in human or experimental animal studies, increased staining for αS Ser129 should not be directly equated to inclusion formation without validation with other non-phospho-specific αS antibodies or other histological analyses.

### Rodent studies of induction and spread of αS inclusion pathology

In the next sections, we discuss the studies utilizing αS transgenic mice and WT (i.e., non-transgenic) mice or rats that have been conducted to try to provide evidence that conformational templating of αS aggregates can occur in vivo, but we also critically highlight some of the technical shortcoming and the complexity in assessing the mechanisms involved in promoting αS inclusion pathology in these studies. In fact, some of the findings from these studies also support that other mechanisms can also be important in driving the spread of αS pathology. Comparing the findings from these studies, utilizing αS transgenic and WT mice also provides important clues to biological barriers that are likely important to naturally suppress the spread of αS pathology. However, it is also important to note that the direct comparison of the findings from different groups can be complicated by differences in experimental conditions, for example the type of PFSPs used, which can be affected by many factors including the sonication conditions used for the preparations.

#### αS transgenic mouse models most commonly used for induction or transmission of αS pathology

Most of the αS transgenic mouse lines that were used to study transmission of αS pathological entities or αS conformational templating mechanisms were identically created with the human αS cDNAs cloned into an expression vector where transgenic expression is driven by the mouse prion promoter [19]. Three of these transgenic mouse lines expressing WT human αS (line M20), A53T human αS (line M83) or E46K human αS (line M47) have been used. These αS transgenic mice predominantly express human αS throughout central nervous system (CNS) neurons [54, 64] as would be expected from expression driven by mouse prion protein promoter [19]. The expression levels in all three transgenic mouse lines are similar but the expression in M20 αS mice is modestly higher than for the M83 αS mice, while the expression in M47 αS mice is slightly lower [54, 64]. Importantly, as the mouse prion protein is not exclusively expressed in neurons, the expression driven by the mouse prion protein promoter occurs not only in CNS neurons, but also at substantial levels in glial cells especially in astrocytes [117, 181].

Native homozygous M83^+/+^ αS mice normally develop a late-onset (8–15 months of age) severe motor phenotype leading to death that results from the formation of neuronal αS amyloidogenic inclusions predominantly throughout the spinal cord, brain stem, thalamus and midbrain areas, and with some αS inclusions in the motor cortex [64]. Remarkably, dopaminergic nigral neurons are resilient to the formation of αS inclusions in these mice despite being surrounded by neurons with αS inclusions [64]. In addition, detailed analyses of the initial presentation of αS inclusions throughout the neuroaxis in these mice, before they are symptomatic, showed that they randomly appear without any clustering or direct evident of general spread [54]. This finding is more consistent with concurrent multifocal formation of αS inclusions with variable spreads as we suggest could explain some of the presentation of Lewy pathology in human brains (see below). Native hemizygous M83^+/−^ αS mice can also develop similar pathologies and phenotype but these are delayed to later than 22 months of age. Native homozygous M20^+/+^ or hemizygous M20^+/−^ αS mice do not develop any overt motor dysfunction or the presentation of αS pathology throughout their normal lifespan [54, 64]. Homozygous M47^+/+^ αS mice naturally develop a similar phenotype and neuroanatomical distribution of αS inclusions as the M83 αS mice but with a later onset (>15 months) and with morphologically distinct αS inclusions which are more round and compact reminiscent of human inclusions [54].

#### Brain extract injection in the cerebrum of αS transgenic mice to model pathogenic transmission

In studies aimed at demonstrating that αS pathology could be transmitted, young M83^+/+^ or M83^+/−^ αS mice were injected in various brain regions with CNS extracts containing αS aggregates from older motor impaired M83^+/+^ αS mice. These injections resulted in both an earlier presentation of αS pathology and motor phenotype [13, 125, 137, 184], and this induction was reportedly not observed in one set of M83^+/+^ αS mice injected with brain extracts generated from healthy M83 αS mice [137]. However, it is important to point out that these studies have not unequivocally demonstrated that αS itself was the inducer of pathology being transmitted as CNS extracts from diseased mice would contain potential inducers of many pathological alterations including elevation in gliosis and various cellular injury response activators. To definitely demonstrate that the induction of pathology was specifically due to transmission of αS molecules, a key experimental control usually includes αS immuno-depletion where the αS present in the sample is removed by antibody absorption. Intracerebral injection of brain lysates from patients with multiple system atrophy (MSA) was also shown to induce αS inclusion pathology in M83^+/−^ αS mice [184], but again αS immune depletion from these extracts was not performed to demonstrate that the presence of αS aggregates was responsible for inducing these changes. In addition, the pattern of αS pathology induced in these studies was typical of the pattern observed in aging M83 αS mice such that the distribution did not appear to follow progression from the injection site [184] or that typically observed in MSA brain. Because clinicopathological differences between Lewy-related cytopathology and MSA are now well recognized [132], it remains to be clarified whether the shared concept of “synucleinopathy” between MSA and PD/DLB is corroborated by such models. Similar experimental passaging studies using CNS extracts from diseased mice into younger health αS transgenic mice to initiate earlier motor phenotype and αS pathology were also recently shown using αS transgenic mice with expression driven by the Thy-1 promoter [165]. Collectively, these studies showed that CNS extracts from αS mice or human brains that are undergoing neurodegeneration can induce pathology in αS overexpressing mice that are inherently primed as internal template but it still remains to be confirmed whether this was due to direct conformational templating by amyloidogenic αS species and subsequent spatial spread. In addition, these studies heavily relied on pSer129 staining.

#### Soluble αS or PFSPs injection in the cerebrum of αS transgenic mice to model pathogenic transmission

A more direct experimental paradigm to try to demonstrate the involvement of αS conformational templating in the induction and spread of αS is the brain injection of in vitro produced recombinant PFSPs. The cerebral injection of recombinant, exogenous PFSPs can induce the formation of intracellular αS inclusion pathology in M83^+/+^ αS mice resulting in the typical motor impairments and paralysis characteristic of the phenotype that occurs in these mice with aging [13, 125, 162]. Furthermore, the observed induced αS pathology was consistent with progression from the brain injection sites used (e.g., hippocampal, cortical or striatal) with the induced brainstem and spinal cord pathology likely reflecting the selective regional vulnerability of these transgenic mice [125, 162]. However, the majority of the white matter staining that was initially reported as an evidence for spread of αS pathology along white matter tracts [125] was later attributed to the cross-reactivity of the antibody anti-phosphorylated Ser129 (pSer129)/clone 81A with phosphorylated NFL [162].

**Fig. 2:**
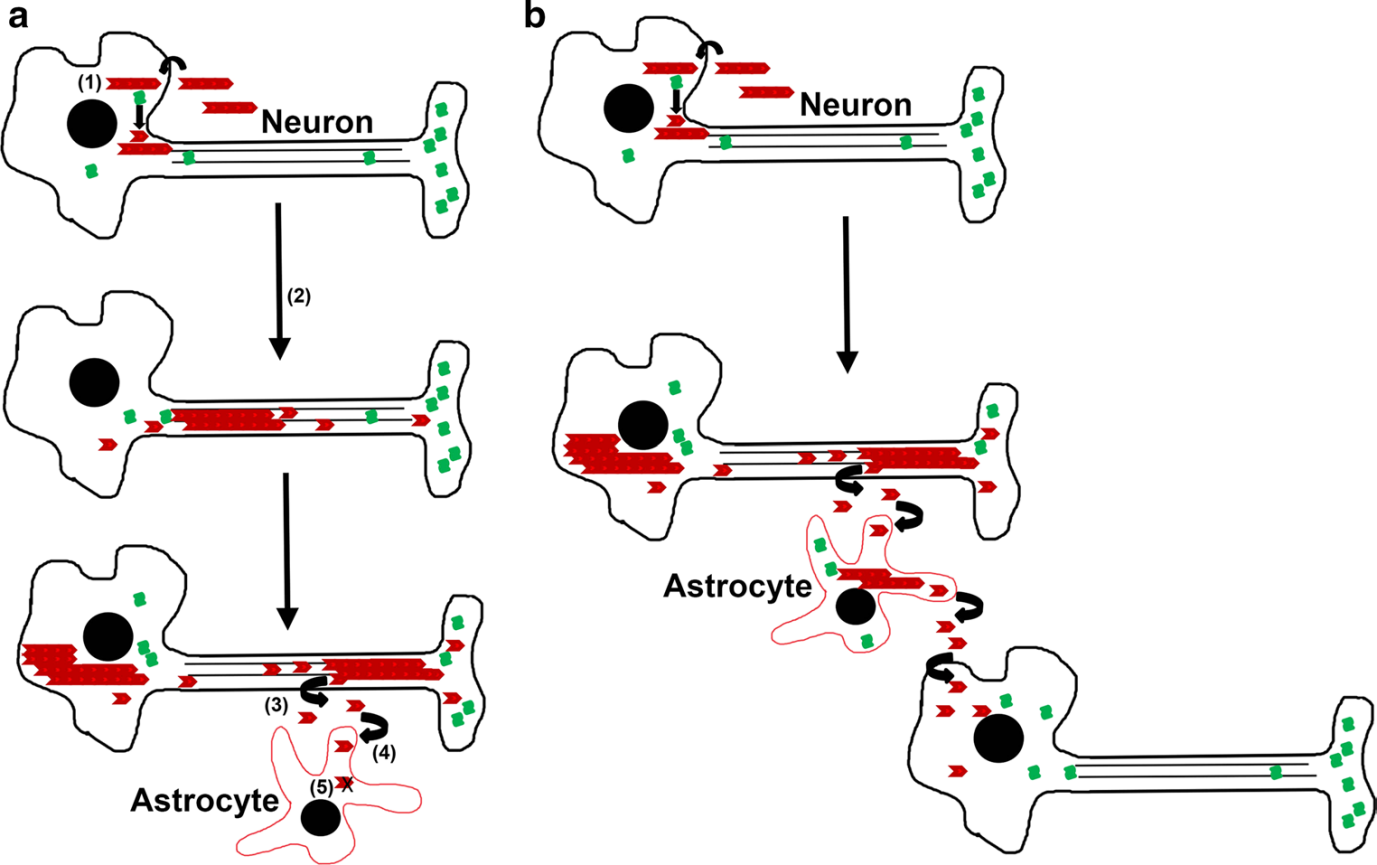
Diagrammatic representation of the mechanisms that can modulate the spread of αS inclusion pathology in WT and αS transgenic mice. **a** The cytoplasmic entry of PFSPs (*red shapes*) followed by the interaction with normally unstructured soluble αS molecules (*green shapes*) can induce their conversion into β-pleated sheet structures (*1*). In this form, αS can elongate into larger amyloidogenic polymers that coalesce into protein inclusions (*2*). Some of the amyloidogenic αS species may be released by neurons into the extracellular space (*3*), but if uptake occurs by glial cells (e.g., astrocytes) (*4*) that do not express αS, this extracellular αS can be terminally degraded (*5*). **b** Similarly, in αS transgenic mice with expression driven by the mouse prion protein promoter, amyloidogenic αS species can also be released by neurons into the extracellular space and taken up by glial cells. However, due to the ectopic expression of αS in transgenic glial cells, this uptake can result in αS inclusion formation that enhances the spread of αS pathology

In similar types of studies, intracerebral injections of PFSPs into adult M20^+/−^ αS mice also resulted in a robust induction of αS inclusion pathology that spread over time from the site of injection [160]. However, cerebral injection of the non-amyloidogenic Δ71–82 αS deletion mutant, which under physiological conditions cannot form amyloid fibrils, directly influence the in vitro polymerization of normal αS or directly induce the seeding of αS inclusions in cultured cells [66, 126, 163, 189], was able to induce αS inclusion pathology, albeit less robustly than PFSPs [160]. In addition, the brain injection of PFSPs in adult M20^+/−^ and M83^+/+^ αS mice resulted in the abnormal accumulations of NFL and peripherin [160], a neuronal intermediate filament protein that can be expressed in the CNS as a result of injury response [9]. Furthermore, cerebral injection of PFSPs into M20^+/−^ or M83^+/+^ αS mice induced the formation of additional protein inclusions immunoreactive for p62/sequestosome, a general marker of inclusion formation, that were not comprised of αS or NFL suggestive of a more general disruption of proteostasis [160]. Robust gliosis was induced from cerebral PFSP treatment and a significant proportion of the induced αS inclusion pathology was observed in glial cells especially astrocytes [125, 160], which are rarely observed in human brain with PD. αS is normally a neuronal protein expressed only at low levels in glia [60, 63, 87–89, 134], so this robust αS pathology in glial cells likely reflects the ectopic, non-neuronal transgenic expression driven in these cells by the mouse prion protein promoter [117, 181]. Glial cells, similar to many other types of cells, can uptake and conceivably degrade exogenous PFSPs or amyloidogenic αS released by cells [108, 111, 154]. Therefore, glial cells can normally act as a barrier to the spread of αS pathology if indeed cellular release and reuptake are required (Fig. 2a). However, in αS transgenic mice created using the mouse prion protein promoter, the ectopic expression of αS in glial cells, especially in astrocytes can result in a more widespread formation of αS inclusion pathology, thus exacerbating the spread of pathological inclusion and injury responses (Fig. 2b). In addition, this propagation of αS inclusion by glial cells does not have to follow neuroanatomical pathways.

For reasons that are still unclear, not every αS transgenic mouse lines demonstrate vulnerability leading to PFSP-induced αS pathology. In parallel stereotactic cerebral injection studies of PFSPs in M47^+/+^ αS mice, these mice were relatively refractory to the spread of αS inclusion pathology induced by PFSPs as αS inclusions were predominantly observed only at the site of injection [162]. Similar findings was obtained using PFSPs comprised of WT or E46K human αS demonstrating that this limited induction of αS was not due to incompatible heterotypic interaction (i.e., cross-species differences) between PFSPs comprised of WT and E46K human αS.

#### Soluble αS or PFSPs injection in the brain of neonatal αS transgenic mice to model pathogenic transmission

Similar to the induction of αS inclusion pathology following the injection of PFSPs in adult M20^+/−^ αS mice, injection of PFSPs in M20^+/−^ αS neonatal mice can induce the formation of αS inclusion pathology [159]. However, depending on the amount of PFSPs injected, the formation of αS inclusion pathology can appear only 8 months after the injection of PFSPs [159]. This finding is remarkable since the presence of the injected PFSPs in neonatal brains could only be detected for 2–4 days [159]. Similar to the studies using adult animals, induction of αS pathology was also observed following the brain injection of Δ71–82 αS in neonatal M20^+/−^ αS mice, although again it was much less robust [159]. Overall, the induction of αS pathology following the neonatal injection of exogenous αS in M20^+/−^ αS mice was slower, less efficient and delayed compared to similar injections in adult mice which is likely due to the lower expression of murine and human transgenic αS in neonatal mouse brains [159], but these findings indicate that the initial brain injection of αS can trigger a slow biological cascade, perhaps partially involving the initial induction of abnormal αS aggregates and conformational templating. These alterations can then result in the formation of mature αS inclusions long after the initial insult. Therefore, αS inclusion formation can be a very slow process that occurs below detectable levels until the aggregates coalesce to a visible size.

#### Peripheral (intramuscular) injection of soluble αS and PFSPs in αS transgenic mice

To further investigate the hypothesis that αS pathology can self-template and spread from the periphery into the CNS via peripheral nerves, PFSPs were injected in the hind leg muscle (either biceps femoris or gastrocnemius) in M83^+/+^, M83^+/−^ or M20^+/−^ αS mice [161]. In addition, an advantage of this simple non-CNS invasive procedure is that it does not alter brain homeostasis as does brain stereotactic surgeries. The intramuscular (IM) injection of PFSPs in M83^+/+^ and M83^+/−^ αS mice resulted in the rapid and synchronized development of hind limb motor weakness associated with robust induction of CNS αS pathology, predominantly in the spinal cord, brainstem and midbrain areas [161]. This pathological changes occurred at ~2 to 3 months following IM injection in M83^+/+^ αS mice and ~3 to 4 months for M83^+/−^ αS mice. IM injections of PFSPs into M20^+/−^ αS mice did not result in any motor phenotype, but induced a progressive accumulation of CNS αS inclusion pathology moving up the spinal cord detectable at 4 months post-injection and increasing thereafter up to 12 months, the last time point assessed [161]. To provide evidence that the induced CNS αS inclusion pathology following IM injection of PFSPs was due to retrograde transport through the sciatic nerve (the major nerve that innervates lower leg muscles), complete transection of the left sciatic nerve was performed 3 days prior to the IM injection of PFSPs in the left gastrocnemius muscle of M83^+/−^ αS mice [161]. The sciatic nerve transection significantly delayed and in some mice perhaps completely prevented the induction of CNS αS inclusion pathology [161]. However, a subset of these mice still developed motor impairment with CNS αS pathology identical to mice without nerve transection. These findings demonstrated that retrograde transport of PFSPs is partially responsible for the induction of CNS αS pathology in this model, but either other routes including perhaps other minor nerves or transport through blood may also be involved in inducing distant CNS αS pathology in predefined fashion. However, IM injection of soluble non-amyloidogenic Δ71–82 αS was also able to induce CNS αS inclusion pathology, albeit less efficiently than PFSPs, indicating that peripheral inoculation of soluble αS must also be able to trigger the formation of CNS αS pathology by a mechanism that most likely does not involve protein conformational templating [161].

#### Evidence for αS pathogenic transmission and αS conformational templating in WT mice

Recasens and colleagues demonstrated that the nigral injection of LB-enriched extracts from the SN of postmortem PD brains can result in the progressive demise of nigrostriatal dopaminergic neurons in mice associated with increased pSer129 staining and diffuse αS aggregates, but clearly defined LB-type inclusions were not observed [152]. In addition, increased levels of pSer129 staining were observed in distal but connected brain regions such as the striatum and motor cortex. The direct involvement of αS inducing these pathological changes was demonstrated in parallel studies with brain fractions lacking αS or injections in αS null mice. The neurotoxic effects of these LB-containing extracts were additionally confirmed in nigral or striatal injection of monkey brain [152].

Cerebral injection of PFSPs can result in the formation of neuronal anti-pSer129 αS-reactive inclusions in WT mice [124, 130, 162, 173], but the extent that this apparent pSer129 αS pathology is due to aggregated, amyloidogenic αS has not been unequivocally established due to the over reliance on anti-pSer129 antibody staining. For example, the cerebral injection of exogenous αS can result in a rapid, robust but transient increase in pSer129/81A staining that is due to increased phosphorylated NFL staining [162]. Similar to the short half-life of PFSPs in neonatal brains, Masuda-Suzukake and colleagues reported that injected PFSPs in adult brains demonstrated a half-life of less than 7 days [130], but about 30 days therefore pSer129-reactive inclusions started to appear [130]. Cerebral injections of brain extracts from patients with αS inclusions were also able to induce pSer129-reactive inclusions in the brain of WT mice. Interestingly, the time lapse between the detection of the PFSPs and the appearance of detectable pSer129-reactive inclusions suggests that some populations of PFSPs remain below detectable levels or that the PFSPs were able to initiate the seeding or slow formation of αS inclusions before being degraded.

Additional studies also suggest that brain αS inclusion pathology induced by the cerebral injections of PFSPs in WT mice appear to spread from the injection site [124, 129, 173]. Again the majority of these data were mainly based on αS pSer129 immunostaining, but in some studies at least a subset of inclusions, but without a thorough parallel mapping of pathology distribution, were confirmed with an antibody to non-phosphorylated αS [124, 173]. This apparent spread of αS inclusion pathology has been attributed to amyloid-like conformational templating. However, it is important to emphasize that a gradient of injected αS away from the injection site, possibly along normal anatomical connections, could lead to the progressive appearance of αS pathology. In this scenario, there could be progressive “seeding” but no true spread except for distribution of the exogenous seeds. Consistent with this possibility, Rey and colleagues have shown that the injection of exogenous monomeric or aggregated αS in mouse olfactory bulb can result in the transport to neurons of neuroanatomically connected brain regions, and here also the injected proteins had a short half-life (less than 72 h), but no induction of αS pathology was observed in these short-term studies [153]. Importantly, these experimental mouse studies also have not definitely addressed the direction (i.e., anterograde versus retrograde) of exogenous αS transport.

Not every PFSPs cerebral injection studies using mice expressing natural levels of αS have supported the notion that this challenge could lead to the induction and spread of authentic αS inclusion pathology. As such, the cerebral injection of PFSPs, which could robustly seed αS inclusion pathology in cultured cells, only induced a very limited amount of αS inclusion pathology in mice that overexpressed tau protein but with normal levels of endogenous αS [75]. Moreover, in another study, the presence of αS inclusion pathology in WT mice following cerebral PFSP injection was limited to the injection site and it actually diminishes over time rather than progressively spreading [162], suggestive of important biological barriers that can thwart the spread of αS inclusion pathology.

The peripheral IM injections of PFSPs in WT mice did not result in the induction of CNS αS pathology [161]. The lack of induction of CNS αS pathology following robust peripheral inoculation with PFSPs was also shown by Masuda-Suzukake and colleagues where the intranasal administration of 80 μg (4 × 20 μg) of PFSPs did not yield CNS pathology even almost 2 years after the exposure [130]. This paucity of CNS αS induction pathology in WT mice following the peripheral exposure to PFSPs is consistent with the normally low level of expression of endogenous αS in the peripheral nerve and the spinal cord in WT mice [63, 161]. This intrinsic low expression of αS in the PNS constitutes another nature barrier to transmission of αS inclusion pathology.

#### Rat studies of induction of αS pathology using exogenous αS

Paumier and colleges recently showed that the unilateral striatal injection of recombinant mouse PFSPs [146] in rats induces the formation pSer129/81A-reactive neuronal aggregates that over time accumulate in areas that innervate the striatum such as the SN pars compacta, amygdala, frontal cortex and insular cortex. As noted above the pSer129/81A antibody can strongly cross-react with other phospho-proteins including NFL. The formation of protein inclusions was documented with other markers such as Nucleoporin 62/sequestosome-1 (p62/SQSTM1), but localization with αS antibodies was weak [146]. Interestingly other brain regions, such as the locus coeruleus (LC) and the raphe, innervating to the striatum did not develop pSer129/81A-positive accumulations, suggesting selective neuronal vulnerability in this process. Despite the findings that pSer129/81A-positive accumulations were only observed in the ipsilateral SN pars compacta, similar bilateral demise of nigral dopaminergic neurons was observed [146] indicating that the injection of mouse PFSP must induce additional pathogenic mechanisms in addition to protein conformational templating since αS inclusion formation was not required to induce the demise of these neurons and the induced toxic mechanism did not follow neuroanatomical pathways. Further supporting this notion is the findings that a similar unilateral striatal injection of soluble, non-aggregated mouse αS can also induce similar pSer129/81A accumulations and the demise of nigral dopaminergic neurons, although the formation of protein aggregates occurred at a slower rate [146]. Interestingly, the number of pSer129/81A-reactive aggregates in the SN pars compacta following the striatal injection of PFSPs decreased with time, despite nigral dopaminergic demise being similar, indicating that the formation of these aggregates can be reversible [146].

Peelaerts and colleagues recently showed that the direct rat nigral injections of PFSPs preparations with different structural properties defined as “fibrils” or “ribbons” were taken up by dopaminergic neurons and induced increased pSer129 immunoreactivity, but without a concomitant demise of nigral dopaminergic cells [147] demonstrating that these αS species were not intrinsically toxic. However, similar nigral injections of PFSPs in rats overexpressing human A53T αS in dopaminergic nigral neurons using rAAV (see below) could exacerbate the neurotoxicity driven by A53T αS overexpression [147]. These studies also reported that some exogenous human PFSPs could be able to cross the blood–brain barrier and enter the CNS after tail vein injections, but this was accomplished using a substantial amount of PFSPs (80 μg total–8 injections of 10 μg each) [147].

### Pathological sequelae of local overexpression of αS through viral delivery

rAAV was used to mediate the expression of human αS in rat vagus nerve and specifically track the temporal and spatial distribution following αS expression [176]. As expected, human αS was initially detected in the medulla oblongata since the vagus nerve is comprised of axons that originate and terminate in the medulla oblongata and upper cervical spinal cord. However, at later time points human αS could also be detected in a limited number of more rostral neuronal projections in the pons, midbrain, and forebrain. Interestingly, no exogenous human αS was found in cell bodies and it was not present in the SN pars compacta. These findings were interpreted as an evidence for transneuronal caudal to rostral spread of αS.

Several studied have used viral vectors (rAAV or lentivirus) to express αS in adult rats’ or monkeys’ nigral dopaminergic neurons to assess the pathological consequences. Expression of human WT, A30P or A53T αS in rat or monkey nigral dopaminergic neurons using rAAV that stably expresses the transgene (>6 months) resulted in substantial and specific demise of these neurons (30–80 % loss), concurrent with the formation of cellular αS cytoplasmic inclusions and dystrophic neurites [99–101]. Similar results were observed when using a lentiviral-based vector system to express αS proteins in rat nigral dopaminergic neurons [122]. However, it is important to note that in these studies αS pathology was exclusively observed in the neuronal populations transduced to express human αS and that no evidence for transmission of αS pathology to other neuronal population was observed despite that they express endogenous αS. Nevertheless, since spread of αS pathology was not a major focus of these studies, such a process could have been unnoticed. These findings further support the notion that there exist potent biological barriers to prevent the inter-neuronal transmission of αS inclusion pathology. It is also possible that the αS aggregates generated in these expression systems are not permissive to transmission.

### Important issues and mechanisms to consider when interpreting experimental induction of αS inclusion pathology in rodents: potential pitfalls of experiments

Potential pitfalls of current experimental paradigms are summarized as follows:

#### Excessive amount of PFSPs

The amounts of PFSPs that were used experimentally to induce αS inclusion pathology in mice and rat are much larger that would normally be locally encountered even if many cells died simultaneously releasing αS aggregates. Thus, it is possible that in the experimental studies using PFSPs the natural barriers to induction and spread of αS induction pathology were artificially overwhelmed beyond concentrations that would normally occur. On the other hand, these studies are modeling human neurodegenerative diseases with changes progressively occurring throughout many years or decades and in aging brains that typically can have multiple proteinopathies likely reducing the normal abilities to cope with protein misfolding.

#### Questionable artificial enhancement of susceptibility

αS transgenic mice are much more susceptible to the induction and spread of αS protein inclusion pathology compared to WT mice [160–162]. Therefore, under normal expression levels induced αS inclusion pathology may not be very robust, suggesting that normal biological activities can cope with even a bolus insult of aggregated protein. The vulnerability of αS transgenic mice could be due to both the overexpression and ectopic, non-neuronal expression of transgenic αS. αS is naturally a highly expressed brain protein, so it is not surprising that transgenic overexpression renders these mice highly primed to the induction of protein aggregation.

#### Bystander contributions other than molecular conformations

**Fig. 3:**
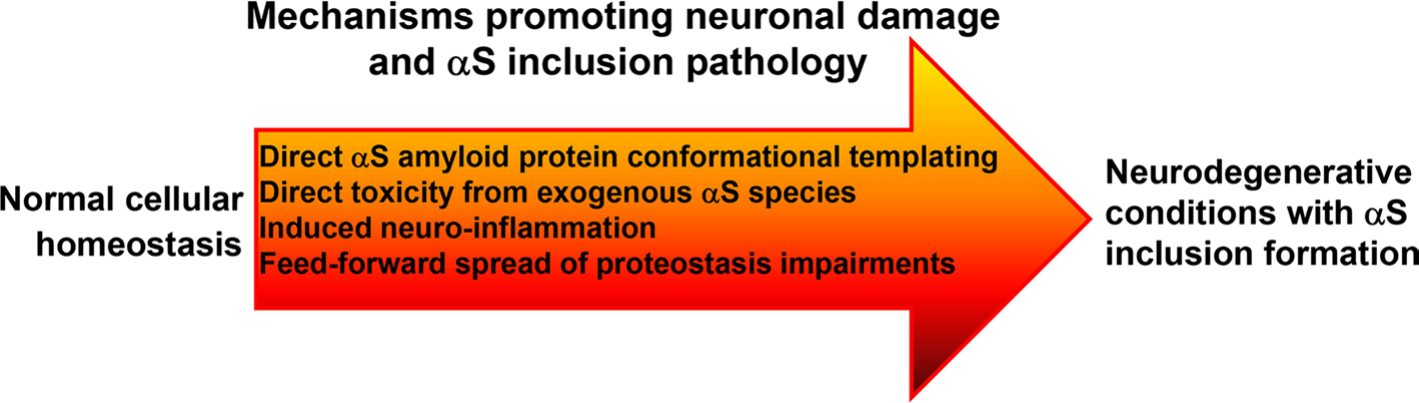
Summary of biological mechanisms that may synergistically promote neurodegeneration with concomitant formation of αS inclusion formation

Although studies using cerebral injection of PFSPs or brain extracts are suggestive of induction and spread of αS induction pathology by protein conformational templating, there are many important issues to consider that make it likely the experimental procedures potentiate this process and that several other mechanisms are involved (Fig. 3). The brain surgical stereotactic procedures used to inject exogenous αS cause both tissue and cellular damage that can greatly promote cellular uptake. Furthermore, it is difficult to completely rule out that some of the observed spread of αS pathology is not simply due to the dispersion of PFSPs in part due to the disruption of cerebral tissue due to needle injections. Once the formation of intracellular αS inclusions have been initiated, these aggregates should actually serve as “magnets” to existing cellular amyloidogenic forms of αS thus hampering the spread of αS inclusion pathology at least until the cell demise, but in the experimental cerebral injection models robust cell death associated with the spread of αS inclusion pathology has not been reported. In addition, the findings that challenges with soluble αS including non-amyloidogenic Δ71–82 αS can also induce αS inclusion pathology in αS transgenic mice and WT rats [146, 159–161] strongly suggest that other mechanisms are involved. If fact, it is possible that the cerebral injection of brain lysates or PFSPs have predominantly demonstrated the initiation of αS pathology due to biological insults.

#### Proteostatic perturbations

The cerebral injection of PFSPs or brain lysates from terminally motor impaired animals could lead to proteostatic perturbations, especially in primed αS transgenic mice thus initiating a progressive cycles of αS accumulation and aggregation. Mechanistically, the two major cytoplasmic protein degradation machineries, proteasome and lysosomal-autophagy, can be inhibited by various forms of aggregated αS [38, 49, 50, 65, 127, 178, 192] that can contribute to the progressive spread of αS pathology. This notion would be consistent with a more global impairment in proteostasis that was noted in one study [160].

#### Glial involvement

The robust activation of gliosis that coincides with the formation of αS inclusion pathology in the αS transgenic models challenged with PFSPs is also suggestive that this process participates in the spread of pathology, either by the aberrant formation of αS in glial cells (Fig. 3) [125, 160] or by inducing inflammatory cellular damage [25, 70, 115]. The original injected PFSPs and additional soluble and aggregated αS released from damaged cells can be potent activators of inflammation [25, 34, 97, 115, 156, 195]. In addition, it should not be overlooked that various forms of exogenous αS could directly lead to cellular toxicity [40, 51, 94, 120], which may also lead to spread of pathology by a positive-feedback process of cellular damage, inclusion formation, and release of αS aggregates.

## Structural template (neuroanatomical considerations)

### Distally accentuated deficit of nigrostriatal and cardiac sympathetic systems: an intraneuronal gradient

In human brains, loss of nigral dopaminergic neurons with LB is the hallmark for PD [73] and explains the deficiency of dopamine in nigrostriatal system. The rate of LB formation is proportional to surviving nigral neurons and precedes their loss [74], although the real role of LB (toxic or protective) in PD remains to be clarified [113]. The number of surviving nigral neurons at the onset of motor deficits of PD is estimated to be 70 % of age-matched control [32]. This estimation is based on an extrapolation from different autopsy series. Deficiency of dopamine at their axon terminals in caudate/putamen is more profound (30 % of age-matched control) [32] than the loss of nigral neurons [85], indicating distally accentuated gradient of degeneration, also confirmed by PET studies in PD patients at the onset of motor deficits. It remains to be clarified what kind of structural changes are responsible for such distally accentuated deficits in the human brains. However, tracing neurite lesion in the human central nervous system is so complex that the identity (dendrite or axons) and the polarity of neurites harboring pathological deposits are not always evident.

Examination of epicardial nerve fascicles provides a unique opportunity to evaluate how cardiac sympathetic nerves, frequently affected in PD in its early stage, are involved in PD. Histological comparison of cardiac sympathetic nerve at different stages from presymptomatic PD (cases with LBs without clinical manifestations of PD, incidental LB disease: ILBD) to full-blown PD demonstrated that αS deposition commences within distal axons at the presymptomatic phase and spreads towards the neuronal perikarya followed by axonal demise [143] (Suppl. Figure 3). The simple morphology and connection of the cardiac sympathetic nerve clarified the anatomopathological temporal substrate with distally accentuated αS deposition followed by axonal depletion. Subsequent loss of noradrenalin terminals is detectable as decreased myocardial uptake of radio-labeled meta-iodobenzylguanidine (MIBG) as a tracer relative to its mediastinal uptake as the reference (H/M ratio). One of the advantages of myocardial MIBG is its quantitative nature. Such a quantitative feature is hardly detected with other indices of autonomic functions because autonomic functions are usually represented as a balance between sympathetic and parasympathetic systems. As a consequence, progressive decline of H/M ratio in PD patients may suggest a progressive depletion of sympathetic axons in the cardiac tissue [142]. Indeed, direct comparison between remnant axons in epicardial nerve fascicles and myocardial uptake of MIBG (H/M ratio) performed premortem in the same patients confirmed their quantitative correlation in a cohort with different stages from ILBD to dementia with LB (DLB) [172].

### Distally accentuated vulnerability in hyper-branched axons as a structural basis to template distal-dominant neurodegeneration

**Fig. 4:**
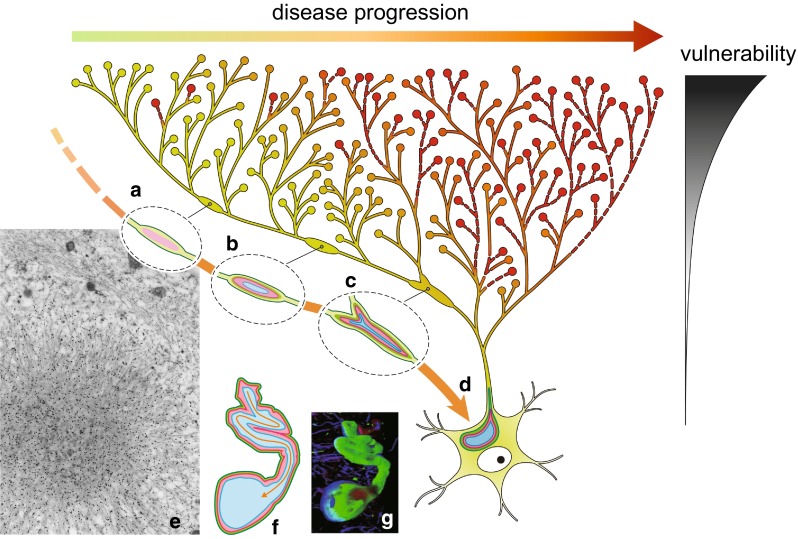
Intraneuronal gradient/progression of Lewy pathology from axon terminals to neuronal soma. Nigrostriatal dopaminergic projections are characterized by long and thin axons with hyperbranching. This structural characteristic enhances distal vulnerability by increasing the length of axons and the number of synaptic terminals, which exponentially enhance the energy burden especially at their distal ends. Normal axon terminals/distal axons (*green*) are gradually filled with αS (*red*) along disease progression as indicated by the *horizontal arrow to the right*. Although it remains to be clarified how such energy expenditure is related to αS deposition, such hyperbranching state enhances the probability of distal axons and terminals to be involved and induces a vicious cycle through enhancing further the progressive compromise in the metabolic support by the decreased number of axons. αS deposition in swollen axons is one of the earliest axonal changes (*pale neurite* *a*). Because deposition of αS is frequent at branching points (Lewy neurites: LNs: *b*, *c*), hyperbranching axons as in the nigrostriatal system may facilitate αS deposition. They spread toward neuronal perikarya, where mature LBs (*d*) are formed. The outermost layer of LBs is composed of neurofilaments (*green* *d*), while the innermost layer is composed of ubiquitin (*blue* *d*) with αS in between (*red* *d*). This three-layered structure, confirmed also by immunoelectron microscopy (*e*) is shared between LNs (*c*) and LBs (*d*). Furthermore, each layer is continuous when a LN is in continuity with a LB (*f*), suggesting that LNs evolve into LBs or Lewy pathology spreads from axon to neuronal soma. (*orange arrow*); *f*, *g* modified from Kanazawa et al. [93]; *e* courtesy, Dr. Masakuni Arima (Director, Komoro Kogen Hospital)

It is proposed that LBs are preferentially formed in projection neurons with long, thin unmyelinated axons [20]. It is expected that energy/metabolic burdens and oxidative stress [80, 169] can be significantly accentuated in these types of neurons [149]. In addition, nigrostriatal axons are characterized by excessive branching, which exponentially enhances these burdens by increasing the number of synapse at the each axon terminal (Fig. 4). Indeed, the number of striatal synapses from a single dopaminergic neuron is estimated to be 102,165–245,103 in rat [131], while it is ten times more abundant to 1,000,000–2,400,000 in human [18], which provides a basis for the distally accentuated vulnerability of nigrostriatal axons. Because some of the genes harboring mutation related to familial levodopa-responsive parkinsonism are related to mitochondria and their functions [128, 136], subsequent energy failure through mitochondrial dysfunction may predispose the nigrostriatal system to degenerate especially in axon terminals regardless of Lewy pathology [128]. Moreover, numerous axon terminals may serve as a portal of entry of external toxins such as 1-methyl-4-phenyl-1,2,3,6-tetrahydropyridine (MPTP) or rotenone to template PD-like symptoms irrespective of Lewy pathology [14]. All of these are compatible with “a mitocentric view of PD” [76, 80].

### Hyperbranching axons as a structural template for Lewy-prone systems and their clinical manifestations

**Fig. 5:**
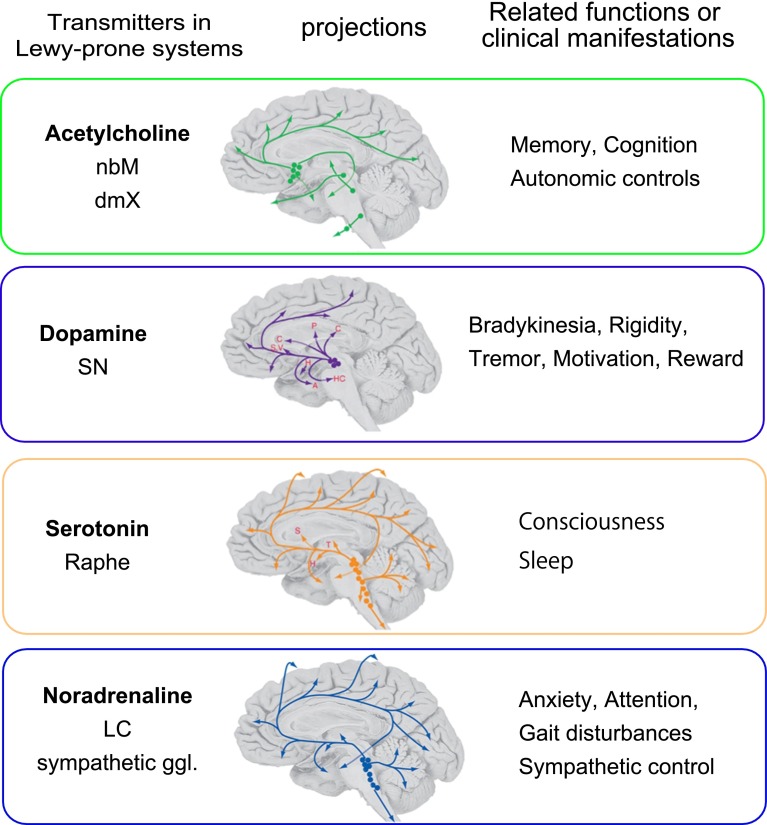
Lewy-prone systems and neurotransmitters. Although the neurotransmitters are different between associated systems, these Lewy-prone systems are characterized by widespread innervation to cerebral cortex, basal ganglia, hippocampus through hyperbranching axons. Such structural template facilitates αS deposition. Such excessive branching of axons is also related to their normal functions or dysfunctions uniformly characterized by non-focal or generalized influences without somatotopy. *nbM* nucleus basalis of Meynert, *dmX* dorsal motor nucleus of vagus, *SN* substantia nigra, *Raphe* raphe nucleus, *LC* locus ceruleus, *ggl*. ganglia; modified from Nolte and Angevine [139] with permission

Lewy pathology is not restricted to the nigrostriatal system but also occurs in a variety of systems with different neurotransmitters such as noradrenergic (sympathetic, LC), cholinergic (parasympathetic, nucleus basalis of Meynert), and serotoninergic (raphe nuclei, raphe) [20, 139] (Fig. 5). Although possible toxicity of dopamine and its metabolites may explain relative accentuation of nigral degeneration, the modality of neurotransmitter is not the major determinant for LB formation. Instead, a unique structural characteristic common to these Lewy-prone systems is hyperbranching of long projection axons which innervate to wide areas in the brain as shown in Fig. 5 [170]. It is likely that this structural template similarly (1) increase the chance to develop αS deposition at these axon terminals, which facilitates αS deposition and (2) this further exacerbates metabolic burden and oxidative stress at axon terminals in these Lewy-prone systems. Centripetal (retrograde) progression of axonal lesion as seen in nigrostriatal and cardiac sympathetic systems may be shared as a rule by these Lewy-prone systems with long projection axons. Of course, this does not necessarily exclude the possibility of intraneuronal spread in the reverse direction, from soma to axon terminals. Indeed, it remains to be clarified whether similar progression along axon is shared in olfactory–amygdala axis [174] or in the striatum, where smaller neurons with shorter axons are intermingled.

From a clinical point of view, such a hyper-branched status of long axon is correlated with their physiological functions and their clinical manifestations without somatotopical presentation. For example, the dopamine deficiency of the nigrostriatal system is related to Parkinsonism and cognition. Cholinergic projections from nucleus basalis of Meynert are related to memory and cognition. Those from parasympathetic nuclei (dorsal motor nucleus of vagus) are related to autonomic regulation. Serotonergic projections from raphe nuclei are related to anxiety, arousal, sleep or nociception. Noradrenergic projections from LC are related to mood, attention and gait. Thalamocortical projection may be related to fluctuation of dementia or hallucination [82]. Such “holistic organization based on large interconnectivity [46]” of Lewy-prone systems is in sharp contrast with the highly organized somatotopy in the primary motor efferent and the primary sensory afferent systems. Such organization is suitable to modulate final motor pathways or primary sensory pathways by exerting more generalized (non-localizationist) influence mainly in an indirect fashion. Consequently, clinical manifestations related to these Lewy-prone systems are vague and generalized such that localizationist recognition of their clinical manifestations is practically difficult.

### Axonal αS spreads toward LB in neuronal soma as in cardiac sympathetic nerve

Although it is established that αS-positive axons appear prior to the formation of LBs [23] (Suppl. Figure 3), it remains to be clarified how Lewy neurites (LNs, mainly in axon) and LBs are related. Three-dimensional analysis of triple-labeled LBs and LNs demonstrated a three-layered structure with neurofilament at the outermost layer, ubiquitin in the core and αS in between [93]. Because each of these three layers is continuous even at the branching point (Fig. 4c) and the junction between LB and LN (Fig. 4d), it is probable that some of LNs in axon may expand to form LB in soma, suggesting again that Lewy pathology extends from neurite to soma to form LB. Furthermore, LNs frequently occur at the branching point of axons (Fig. 4c), it is likely that some local environment related to branching, such as trouble in transport, may predispose αS deposition at each branching point [92]. Although the exact mechanisms for αS deposition at distal axon/synapse [10, 57] and for the preferential deposition at branching points still remain to be clarified, all of these findings are compatible with the observation that spread of axonal αS deposition is followed by axonal depletion to yield the so-called distal-dominant degeneration. Because hyperbranching of axons may predispose not only axon degeneration but is associated also with αS deposition, it is plausible that such structural characteristics may feature selective vulnerability of all Lewy-prone systems including peripheral sympathetic systems as their “structural template”. Distal axons could be dysfunctional or depleted early in the disease course even when their respective neuronal cell bodies remain relatively intact (intraneuronal gradient). Awareness of such early neurite pathology before neuronal death [32, 119] will require the revision of the cell death-oriented paradigm of neurodegeneration to “neurite pathology” or “nexopathy”, so that earlier and more relevant to clinical manifestations are detected for early therapeutic intervention [27, 183].

### Early autonomic involvement in PD

Autonomic nervous system outside the CNS serve as direct effectors (vasomotor, sudomotor, pilomotor, visceromotor) or includes primary sensory (viscerosensory) components. They are also characterized by unmyelinated hyperbranching axons and therefore frequently harbor αS deposits. Because cardiac sympathetic nerves could be affected even in asymptomatic patients with incidental LBs in the CNS [143] and there are some cases with αS deposits exclusively in cardiac sympathetic axons without LB in the CNS [133], it is plausible that cardiac sympathetic nerves are one of the initial sites of Lewy pathology involvement. Some peripheral autonomic axons and ganglia are accessible through skin [47] or gastrointestinal biopsies, which can be used to try to predict the presence of CNS LBs [83]. More extensive histological comparison of CNS, spinal and peripheral autonomic system for αS in an autopsy cohort of 98 patients without clinical manifestations of PD demonstrated frequent involvement of spinal cord and peripheral autonomic nerves in parallel with olfactory bulb and dmX [15]. To address how these autonomic involvements by αS is related to PD, initially asymptomatic patients harboring chance identified αS deposits in surgical specimens of abdominopelvic organs were followed up for 30 months. Because UPDRD-II scores of these patients were higher and their myocardial uptake of MIBG (H/M ratio) was lower than in αS-negative controls, it is likely that such αS deposition in neurons of abdominopelvic organs represents one of the earliest stages of PD [135] or pure autonomic failure (PAF) [77]. Unexpected spreads of αS deposits including autonomic systems are not uncommon [7].

### Possible discrepancies between αS deposition, neuroaxonal depletion and clinical manifestations

Since initial axonal accumulation of αS can be followed by loss of axons, the absence of αS deposits could represent either the absence of Lewy pathology or axonal depletion after formation of Lewy pathology or by other causes such as ischemia. Thus, parallel evaluation of neuroaxonal components is necessary [4, 143]. Depletion of sympathetic axons in the absence of αS deposits can suggest the presence of Lewy pathology elsewhere, unless otherwise explained by ischemia, for example. However, the absence of αS deposits may indicate either the total absence of Lewy pathology or loss of neuron after formation of Lewy pathology [74]. Therefore, αS IHC not assisted by evaluation of neuroaxonal components could be misleading. Additional attention should be paid on possible influences of staining procedures and anti-αS antibodies, which may greatly alter αS immunoreactivity [2, 105, 148, 185]. Anyway, abundant αS inclusion pathology in dmX may not necessarily be correlated with more profound neuronal depletion, while evident neuronal loss in the SN is not necessarily correlated with abundant αS deposits, which should be taken into account when considering regional gradient or lesion spread. Since distal axon and synapse are preferentially affected in LB disease before involvement of neuronal soma, it is possible that apparent preservation of neuronal soma does not assure the functional integrity. Therefore, discrepancies between αS deposition, neuroaxonal depletion and clinical manifestations could be trivial and troublesome, which should be carefully correlated for sound interpretation. It remains to be clarified whether such discrepancies may be further enhanced or complemented by possible participation of αS oligomers, now selectively detectable with proximity ligation assay [155] or synaptic αS detectable with PET blot [106] in human brain. Finally, clinicopathological interpretations may be hampered by coexisting lesions other than αS [86, 104].

In summary, Lewy-prone systems are structurally characterized by long hyperbranching axons that template distal-dominant degeneration with αS deposits. This degenerative process predominantly starts at distal axons and spreads in retrograde direction towards neuronal soma. This structural feature with barely somatotopic feature is also related to clinical presentations of PD/DLB. How these building blocks are organized into an order in this disorder is discussed below.

### Possible mechanism for regional gradient (dmX > LC > SN) based on single hit hypothesis

As initially noted by Lewy, LBs in the dmX are usually more abundant than those in SN or LC in PD patients. This regional gradient (dmX > LC > SN) was systematically demonstrated by Del Tredici and Braak [20, 45] based on a series of PD cases with LBs in dmX. Because their careful observations were based on serial thick sections to encompass the entire target nuclei, their irrefutable data are much more reliable and convincing than chance observation on routine single section used in other studies. Furthermore, this apparent gradient in regional hierarchy is consistent with a hypothesis that αS deposition is always initiated at dmX and subsequently occurs in LC and finally in SN. Such translation of this stereotyped regional hierarchy into a predefined chronological unidirectional sequence provided a basis to construct a staging framework of PD based on αS deposition. As a more mechanistic interpretation, this regional gradient is interpreted as a possible spread of αS deposition from the dmX to LC then SN along neural circuit, as if propagating transsynaptically. This spatiotemporal axis proposed by this conceptualization greatly improved our understanding of PD by displaying its clinicopathological sequence on a defined timetable.

### From single hit to multiple hit hypothesis

In addition to this putative upward progression of αS lesion from the lower brainstem to upper brainstem, independent involvement of olfactory–amygdala pathway by αS has been noted [81]. Although direct dopaminergic projection from SN to olfactory bulb was recently identified in rats [84], these two putative axes have been considered not contiguous inside the human brain. One of the alternative explanations for their co-occurrence is that some infectious agents, like virus, may initiate olfactory involvements through nasal cavity and vagal involvements through alimentary tract in parallel. This explanation with virus-like agent, however, awaits confirmation by identifying possible infectious agents. Nonetheless, the concept of this “dual-hit” hypothesis suggests a possibility that initiation of αS deposition is not necessarily monofocal and could be induced in multiple independent systems by some factors outside the neuraxis. How these Lewy-prone systems are related or not is one of the major issues in this series as discussed below.

### Is the distribution of Lewy pathology sufficiently hierarchal to corroborate a unified hypothesis of unidirectional spread?

Typical gradient of Lewy pathology (dmX > LC > SN), initially proposed by Del Tredici and Braak [20, 45], is observed in some of (but not all) the cases in other series [78]. Data on this gradient in human brain are, however, still highly conflicting and cases with LB pathology not in agreement with this gradient are being accumulated [8, 26, 90, 91, 144]. Moreover, exceptional cases with LBs in cerebral cortices with less involvement in the brainstem have been reported [103, 193], suggesting that initiation at the lower brainstem is not a prerequisite for development of LB pathology. Additional exceptions include (1) LBs only in cardiac sympathetic nerve [133], (2) their selective appearance only in LC [123], (3) their selective appearance in the autonomic system as in PAF [77] and (4) REM sleep behavior disorder (RBD) which is frequently associated with Lewy pathology [17] and could be a prodrome long before the development of overt motor deficits of PD type. Although anatomopathological substrates for RBD remain to be clarified [16, 48], it is likely that Lewy pathology different from typical PD which may be related to this peculiar clinical manifestations. Although such focal and isolated LB depositions are not in accordance with the typical gradient (dmX > LC > SN), these could be preludes to full-blown PD or DLB. Importantly, parkinsonism is not a necessary feature for these exceptional cases. These focal presentations of LBs could be termed “focal LB disease”, suggesting possible independent development of Lewy pathology in different Lewy-prone systems. Because such “non-motor” symptoms are clinically detectable even before the appearance of parkinsonism, how to take these “unpredictable” clinical features, possibly related to Lewy pathology, into account of diagnosis and management is one of the key issues in the clinical field. Nevertheless, the clinical and pathological heterogeneities indicate that the involvement of Lewy-prone systems is not exclusively stereotyped as to corroborate its unidirectional spread.

### Possible interneuronal connections between Lewy-prone systems to explain disease evolution

**Fig. 6:**
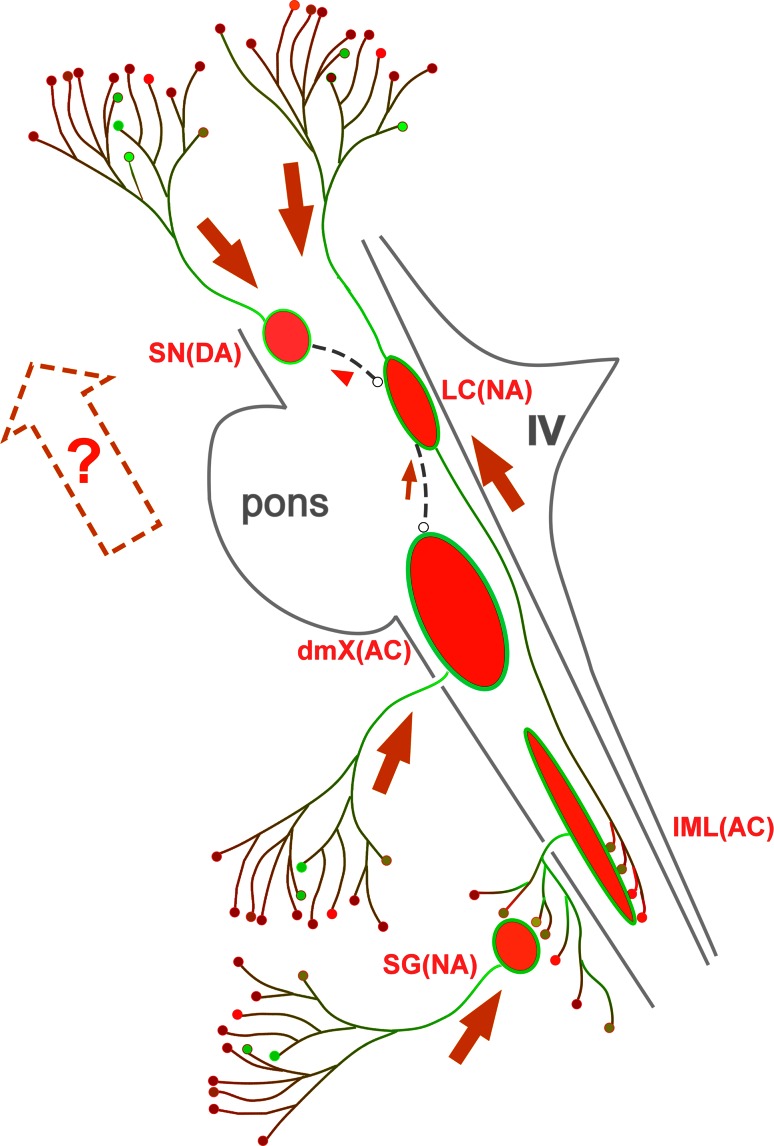
Intraaxonal progression of Lewy pathology in Lewy-prone systems. αS pathology is initiated at distal axons and spreads toward neuronal cell bodies in each Lewy-prone system as indicated by *orange arrow*, respectively. This axonal spread is in parallel with the direction of the so-called the “*upward spread*” (*broken* and *empty arrow* containing “?”) up to LC. However, the direction of this axonal spread is “*downward*” above LC. Additional projections form SN to LC (*gray interrupted line*) and that from LC to dmX (*gray interrupted line*) may be one of the candidate structures that may mediate transneuronal spread from dmX to LC (*small arrow in orange*) and that from LC to SN (*arrowhead in orange*), while these have not yet been documented in human brains with LB disease. *DA* dopamine, *NA* noradrenalin, *IV* forth ventricle, *AC* acetylcholine, *SG* sympathetic ganglia, *IML* intermediolateral nucleus

Even though distribution of LB pathology could be heterogeneous, there are cases with typical gradient of Lewy pathology (dmX > LC > SN) as formulated by Braak and Del Tredici. Even though brainstem nuclei are interconnected with each other directly or indirectly [44], it is not yet clarified which anatomical pathways may provide solid infrastructures to facilitate spreads of αS lesions across the systems from dmX, LC to SN in the human brainstem. It is worth considering how such gradient could be generated. As shown in Fig. 6, each of these systems is mainly equipped with major efferent projections with hyperbranching axons: upward projection from SN; downward projection from dmX; and both up- and downward projections from LC. In each system, αS lesion spreads retrogradely along these hyperbranching axons from axon terminal to neuron (Fig. 6, large orange arrows). If such intraneuronal progression along these projecting axons is unidirectional in each system (dmX, LC, SN), these intraneuronal spreads are not sufficient to explain the upward progression from dmX, LC, to SN, which may require additional transsynaptic transmission. If axonal spread of αS deposit is retrograde and descending as shown in Fig. 6 (descending orange arrows to SN and LC), it is still necessary to explain the ascending αS pathology by other pathways. Additional projections form SN to LC (Fig. 6, gray interrupted line) and that from LC to dmX (Fig. 6, gray interrupted line) may be one of the candidate structures that may mediate transneuronal spread from dmX to LC (small arrow in orange) and that from LC to SN (arrowhead in orange). Even though such downward projections [182] from SN to LC [29] and those from LC to dmX [190] have been reported, it is not yet clarified whether these candidate or other connections are neuroanatomical substrates to facilitate predictable and stereotyped spreads of αS aggregates between these LB-prone nuclei. Although complete neuroanatomical map for possible networks between these brainstem nuclei is far beyond the scope of this review, more detailed studies to demonstrate contiguous αS lesion, if any, by tracing along these candidate structures may reinforce the hypothesis of transneuronal spread by way of axon. Otherwise, morphological substrates for stereotyped progression of αS remain hypothetical in the human brain.

### Could direction of intra- and interneuronal spread of αS be reversible?

**Fig. 7:**
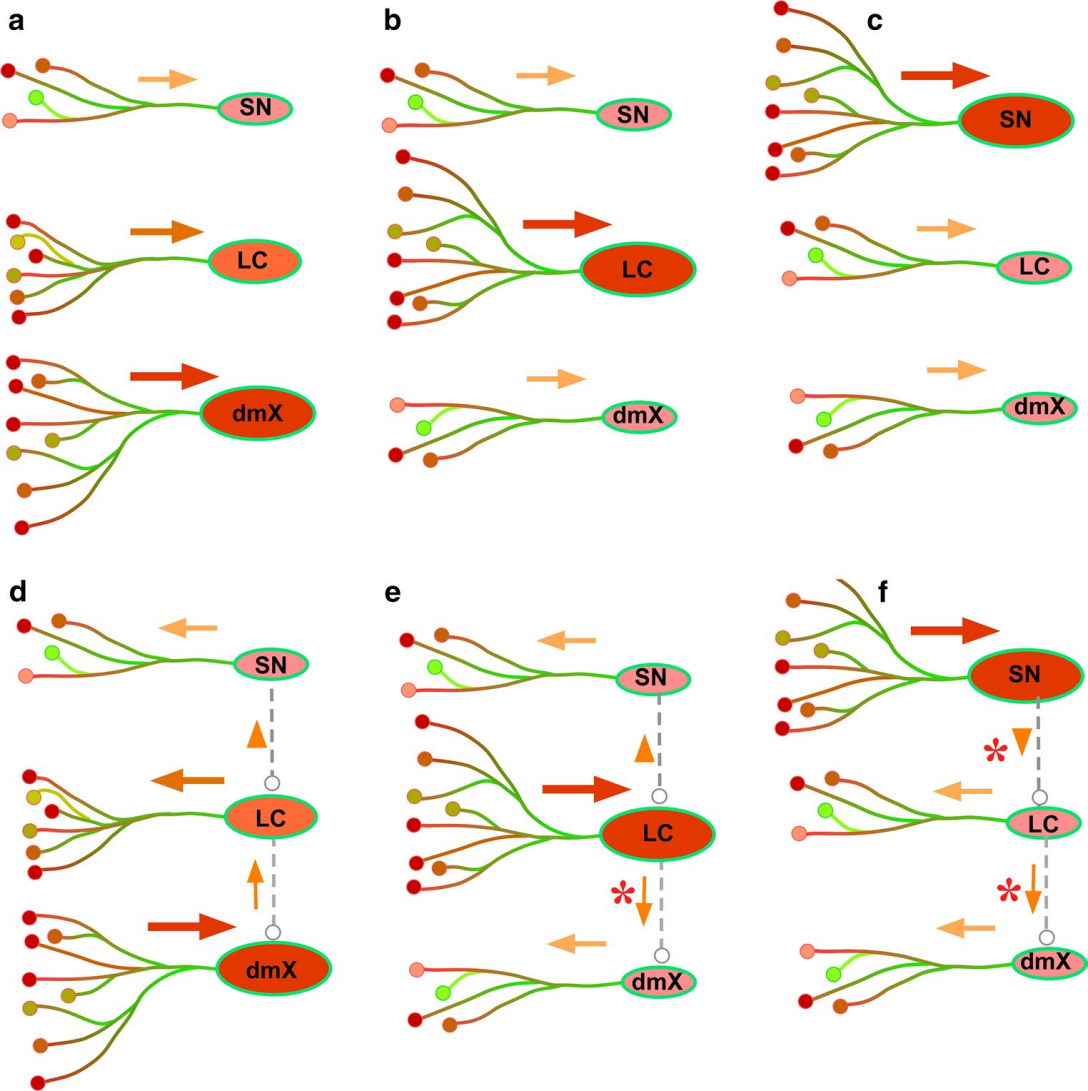
Non-connected or connected explanations for variable gradient. Relative quantity of Lewy pathology could be quite variable with predominance at the dorsal motor nucleus (dmX, **a**, **d**), the locus coeruleus (LC, **b**, **e**) or the substantia nigra (SN, **c**, **f**). Putative directions of intraneuronal spread for αS inclusion pathology are indicated with *orange* or *red arrows*. Expected directions of possible transneuronal spread of αS inclusion pathology along interrupted lines are indicated with *gray arrows* and *arrowheads*. Either dmX (**d**), LC (**e**) or SN (**f**) is assumed to be initially involved and subsequently spread of αS inclusion pathology through interneuronal connections. Both intra- and inter-neuronal directions are reversed according to the primary site of involvement (**d**–**f**). See text for details

Even if the connections discussed above would be available, this explanation may be faced with difficulties as shown below. Figure 7a, d shows the typical gradient of Lewy pathology (dmX > LC > SN). As fully discussed above, intraneuronal progression starts distally and spreads axonally towards neuronal soma (Fig. 6, orange arrows) in each system. If this regional gradient is mediated by transneuronal spread from dmX to LC (Fig. 7d, interrupted lines with arrow), aggregated αS in the dmX may be taken up by axon terminals and then spreads to the soma of LC neurons. Following transneuronal transfer of aggregated αS to LC neurons, it is expected to spread from soma of LC neurons to their distal axons, which is in the direction opposite to what would be occurring in Lewy-prone systems.

Figure 7b, e shows atypical gradient of Lewy pathology (dmX < LC > SN). This time, LC neurons are initially involved from its distal axons as expected. When this αS pathology in LC neuron spreads to dmX (Fig. 7e), however, the direction of transneuronal spread is reversed (Fig. 7e, asterisk). It remains to be clarified whether the same axonal connection between dmX and LC could serve as bidirectional conduit or alternative connections are necessary to facilitate this reverse transmission. In either case, transport of αS aggregates to dmX soma may induce intraneuronal spread from soma to axon (Fig. 7e, orange arrow). If the regional gradient of αS lesions of human brain is explained by interneuronal transmission (Fig. 7d–f), the direction of intraneuronal and that of interneuronal transmission would have to be drastically reversed from a case to another. Although unified hypothesis of conformational template-like propagation may explain spread of αS lesion and its gradient, we have no unified mechanism to explain how neuroanatomical routes and direction of αS spread could be so variable at least in the human brain.

In contrast, Fig. 7a–c shows typical and atypical gradient of LB pathology. Intraneuronal spreads from axon to soma are shared among dmX, LC and SN regardless of the primary site of involvement. Note that this scheme is achieved only when interneuronal connections (interrupted lines in Fig. 7d–f) are eliminated. Because the underlying structures used as anatomopathological substrates for interneuronal connections are not consistent, is it plausible to speculate that each Lewy-prone system could be more or less independent rather than being connected in a predefined order? This hypothesis based on “relative likelihood of different brain regions to manifest Lewy pathology [26]” may better explain why Lewy pathology may not necessarily be in conformity with the ascending spread in the brainstem or sometimes could even be isolated as “focal LB disease”. In other words, because the regional abundance of αS is not homogeneous in the human brain, the relative abundance of αS inclusion pathology (dmX > LC > SN) is not necessarily consistent with a chronological scheme such as staging. Such awareness based on this more flexible hypothesis, in turn, may lead us to reconsider the framework of clinical diagnosis of PD to enhance the clinical recognition of the wider spectrum of LB disease not restricted to PD or DLB [11, 12]. As described above, αS deposition in some parts of the human brain starts at axon terminals and subsequently spreads along projecting axons toward cell bodies. Unified spread of Lewy pathology from lower brainstem to upper brainstem may only be observed in some selected patients. However, the underlying neuroanatomical map to assure this unidirectional spread is not yet apparent. It is neither demonstrated whether the spread of αS lesion could be bidirectional (from axon to soma or from soma to axon) in human brain.

Alternatively, or more simply, Lewy pathology at least in the human brain could develop independently even without direct spread between contiguous structures. Such “system-autonomous” development of Lewy pathology is related to the shared “structural template” of thin unmyelinated hyperbranching axon structures that facilitates LB pathology (Figs. 4, 5), which provides the basis for the concept of “focal LB disease”. Because development of Lewy pathology is facilitated more or less by such “structural template”, gradients in lesion density could be determined by the relative propensity dependent on underlying structures (degree of divergence of axon, for example), influence of mitochondrial stress, or energy expenditure.

Of course, this system-autonomous explanation for LB pathology and its regional gradient does not exclude the possibility that transneuronal spreads of αS deposits and its uptake through neuronal soma are also taking part in human brain with PD. Currently, however, direct evidence to support this transneuronal spread and uptake is not yet clear in human brain with sporadic PD. Even without such transneuronal transmission, gradient of αS lesion and its variations could be well explained by independent development of αS lesion in different systems not necessarily linked with each other.

## Conclusions

Many studies and reviews on the topic of neurodegenerative diseases with LBs are organized as if “molecular template” for αS and its spread along “structural template” are unequivocally coupled to provide a direct mechanism to explain the progressive clinical and pathological nature of these disorders. This coupling of molecular and structural templates may be interesting, convenient and even relevant if both aspects share common molecular processes as at least partially demonstrated in several experimental studies. However, other aberrant mechanisms including neuroimmune activation, injury responses and/or general perturbation of proteostasis, which can also result in promoting the spread of αS pathology, have also been implicated in these experiments. Furthermore, a direct association between molecular and structural templating as a general unified mechanism for spread of disease along neuropathological pathway would require a strictly directed inter-neuronal transmission (e.g., transsynaptic) of αS aggregates, but at this time the current experimental studies do not explicitly support this notion. Although this coupling may partially explain stereotyped regional gradient of αS lesions in human brain, frequently accentuated in lower brainstem and its upward progression, this simplified hypothesis is not sufficient to account for the variable distribution of αS lesions in human brain ranging from focal LB disease to diffuse LB disease. Multifocal formation of Lewy pathology with variable spreads may better explain the independent occurrence of Lewy pathology in different Lewy-prone systems as “multifocal LB disease”. In addition, axonal hyperbranching in combination with other intrinsic cellular properties affecting the abilities of various neurons to cope with misfolded, aggregated αS could result in neuronal populations with different and unique inherent propensities to internally template αS amyloid formation. These inherent differences in individual neuronal populations also could partially explain some of the disordered presentation of αS inclusion pathology in human brains. In contrast with the disordered regional distribution, intraneuronal distribution of Lewy-related degeneration is consistently accentuated at distal axons and retrogradely spreads in an ordered fashion, which is predisposed by their hyperbranching as in the nigrostriatal axons. This order in retrograde spread along hyperbranching axons even in disordered regional distribution of αS of human brain is representative of Lewy pathology. Awareness of this human brain-oriented view is necessary and helpful not only in modeling the αS disorders in animals but also in improving clinical practice for better diagnosis and therapy.

## Electronic supplementary material

Supplementary material 1 (JPEG 594 kb). Suppl. Figure 1: Immunocytochemical analysis of the specificity of pSer129 αS antibodies in detecting αS pathological inclusions in human and mouse brains. Representative images of αS inclusion pathology in the hippocampus of a DLB patient stained with anti-αS antibody SNL4 (a) or anti-αS pSer129 antibodies EP1536Y (b) or MJF-R13 (8–8) (c). Staining with anti-αS pSer129 antibodies EP1536Y of the brain stem region of a symptomatic 15 month old M83^+/+^ αS mouse (M83) with αS inclusion (d), compared to a WT mouse (e) and an αS null mouse (f). Staining with anti-αS pSer129 antibodies MJF-R13(8–8) of the brain stem region of a symptomatic 15 month old M83^+/+^ αS mouse (M83) with αS inclusions (g), compared to a WT mouse (h) and an αS null mouse (i). Inset in i shows the non-specific staining of antibodies MJF-R13(8–8) for the cell bodies and processes of Purkinje cell in the cerebellum of an αS null mice likely due to the non-αS cross-reactively identified biochemically in Suppl. Figure 2. Arrows indicate Lewy pathology in the DLB patient. Bar = 100 μm

Supplementary material 2 (PDF 1645 kb). Suppl. Figure 2: Biochemical and immunoblot analysis of the specificity of several pSer129 αS antibodies. (a) Recombinant human αS was untreated (-) or reacted with casein kinase 2 (CK2) in vitro and analyzed by Western blotting with total αS antibody Syn204 or pSer129 antibodies 81A, EP1536Y and MJF-R13 (8–8). 100 ng of αS protein was loaded in each lane. (b-j) Assessment of the specificity of pSer129 αS antibodies by immunoblot analyses of biochemically fractionated mouse nervous tissues without or containing αS inclusions. (b-f) Mouse brain stem and spinal cord or (g-j) cortex from an αS null mouse, WT mouse, a 2 month old non-sympomatic M83^+/+^ αS mouse (M83) and a 12 month old motor impaired M83 αS mouse (M83-I) sequentially extracted as previously described with solution with increased protein solubility [54]. 20 μg of total protein extracts from the high-salt (HS) and high-salt Triton X-100 (HS/T) fractions and 10 μg from the SDS-urea (SDS/urea) fractions were loaded onto 13 % polyacrylamide gels as indicated above each lane. Western blot membranes were probed with human αS antibody Syn 211 (b, g), pSer129 antibodies 81A (d, h), EP1536Y (e, i) and MJF-R13 (8–8)(f, j), or anti-NFL antibody NR4 (c) as indicated above each blot. The protein bands corresponding to αS and NFL are indicated. The accumulation of aggregated, phosphorylated Ser129 αS is demonstrated in SDS-urea fraction from the brain stem/spinal cord of motor impaired M83^+/+^ αS (M83-I) mice. Non-αS protein bands cross-reacting with antibody pSer129/MJF-R13 (8–8) in the cortex are indicated by asterisks. The mobilities of molecular mass markers are shown on the right

Supplementary material 3 (PDF 307 kb). Suppl. Figure 3: Intraneuronal gradient/progression of Lewy pathology in the cardiac sympathetic nervous system. αS aggregates abundantly accumulate in the distal axons in incidental LB disease (ILBD) at its early phase (early ILBD), which gradually diminish in at its later phase (late ILBD) and disappear in PD, when distal axons are depleted (dotted line). In contrast, αS aggregates progressively accumulate in paravertebral ganglia. Such changes are absent in multiple system atrophy (MSA) and normal controls. From Orimo et al. (2008) [143] with permission
