# Supplementary figures and images for: Propagation of alpha-synuclein pathology: hypotheses, discoveries, and yet unresolved questions from experimental and human brain studies
Source: Acta Neuropathol. 2015 Oct 7;131:49–73. doi: 10.1007/s00401-015-1485-1 (PMC4698305; doi:10.1007/s00401-015-1485-1)

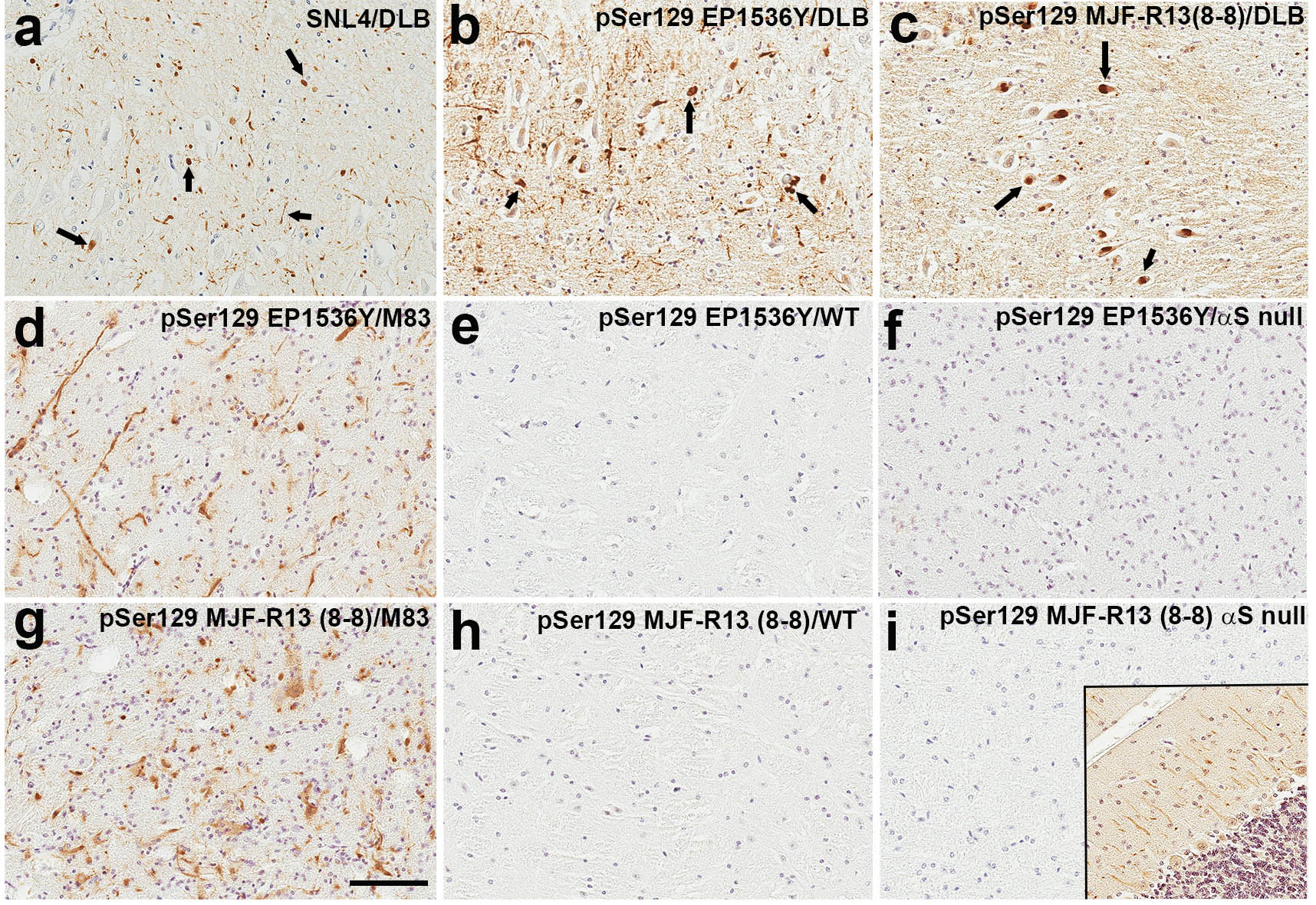

Supplement: Supplementary file 1 — Supplementary material 1 (JPEG 594 kb). Suppl. Figure 1: Immunocytochemical analysis of the specificity of pSer129 αS antibodies in detecting αS pathological inclusions in human and mouse brains. Representative images of αS inclusion pathology in the hippocampus of a DLB patient stained with anti-αS antibody SNL4 (a) or anti-αS pSer129 antibodies EP1536Y (b) or MJF-R13 (8–8) (c). Staining with anti-αS pSer129 antibodies EP1536Y of the brain stem region of a symptomatic 15 month old M83+/+ αS mouse (M83) with αS inclusion (d), compared to a WT mouse (e) and an αS null mouse (f). Staining with anti-αS pSer129 antibodies MJF-R13(8–8) of the brain stem region of a symptomatic 15 month old M83+/+ αS mouse (M83) with αS inclusions (g), compared to a WT mouse (h) and an αS null mouse (i). Inset in i shows the non-specific staining of antibodies MJF-R13(8–8) for the cell bodies and processes of Purkinje cell in the cerebellum of an αS null mice likely due to the non-αS cross-reactively identified biochemically in Suppl. Figure 2. Arrows indicate Lewy pathology in the DLB patient. Bar = 100 μm [file 401_2015_1485_MOESM1_ESM.jpg]

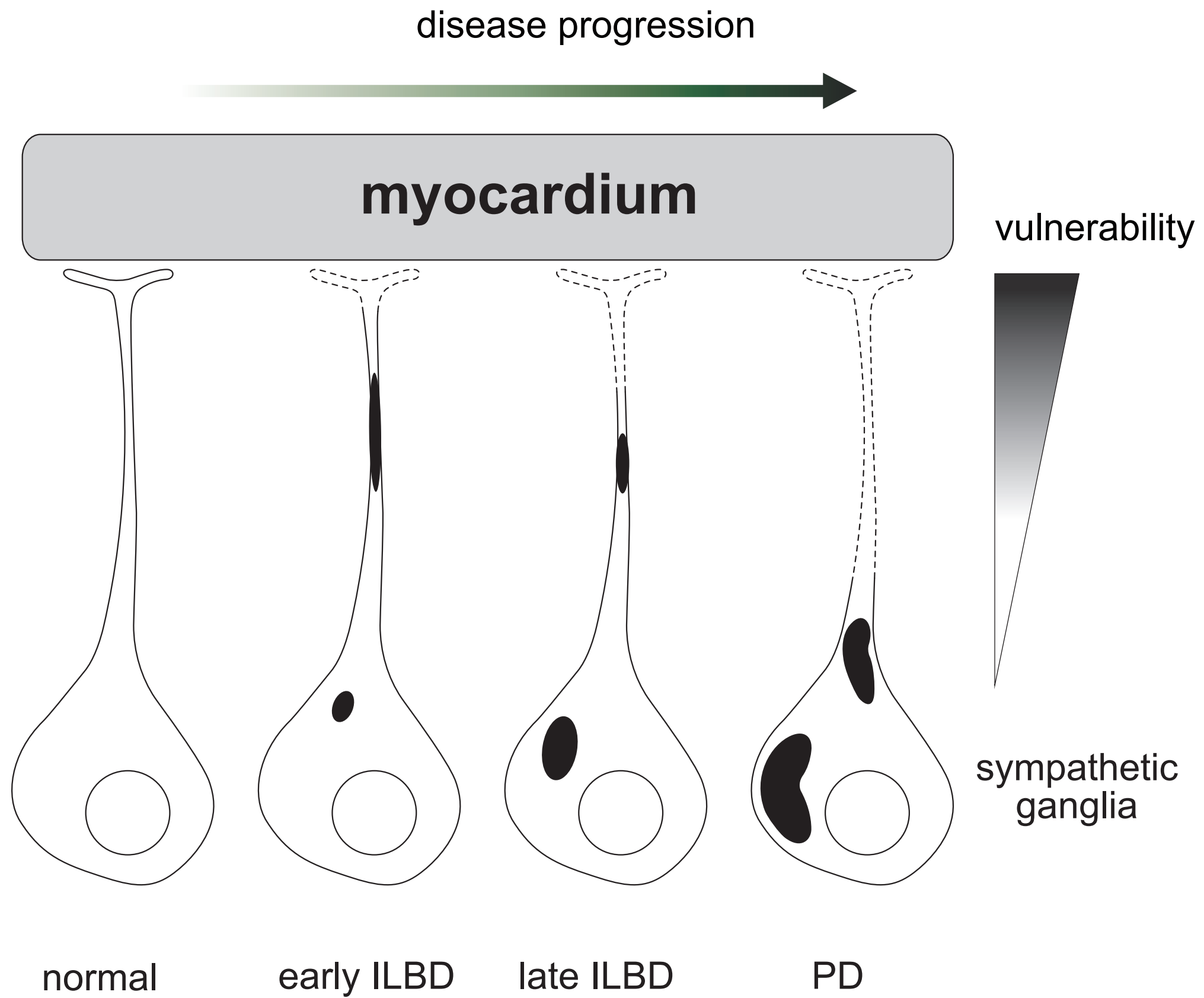

Suppl. Fig 3

**myocardium**

**vu**

Supplement: Supplementary file 3 — Supplementary material 3 (PDF 307 kb). Suppl. Figure 3: Intraneuronal gradient/progression of Lewy pathology in the cardiac sympathetic nervous system. αS aggregates abundantly accumulate in the distal axons in incidental LB disease (ILBD) at its early phase (early ILBD), which gradually diminish in at its later phase (late ILBD) and disappear in PD, when distal axons are depleted (dotted line). In contrast, αS aggregates progressively accumulate in paravertebral ganglia. Such changes are absent in multiple system atrophy (MSA) and normal controls. From Orimo et al. (2008) [143] with permission [file 401_2015_1485_MOESM3_ESM.pdf]
