# Supplementary material for: Propagation of alpha-synuclein pathology: hypotheses, discoveries, and yet unresolved questions from experimental and human brain studies
Source: Acta Neuropathol. 2015 Oct 7;131:49–73. doi: 10.1007/s00401-015-1485-1 (PMC4698305; doi:10.1007/s00401-015-1485-1)
Supplement: Supplementary file 2 — Supplementary material 2 (PDF 1645 kb). Suppl. Figure 2: Biochemical and immunoblot analysis of the specificity of several pSer129 αS antibodies. (a) Recombinant human αS was untreated (-) or reacted with casein kinase 2 (CK2) in vitro and analyzed by Western blotting with total αS antibody Syn204 or pSer129 antibodies 81A, EP1536Y and MJF-R13 (8–8). 100 ng of αS protein was loaded in each lane. (b-j) Assessment of the specificity of pSer129 αS antibodies by immunoblot analyses of biochemically fractionated mouse nervous tissues without or containing αS inclusions. (b-f) Mouse brain stem and spinal cord or (g-j) cortex from an αS null mouse, WT mouse, a 2 month old non-sympomatic M83+/+ αS mouse (M83) and a 12 month old motor impaired M83 αS mouse (M83-I) sequentially extracted as previously described with solution with increased protein solubility [54]. 20 μg of total protein extracts from the high-salt (HS) and high-salt Triton X-100 (HS/T) fractions and 10 μg from the SDS-urea (SDS/urea) fractions were loaded onto 13 % polyacrylamide gels as indicated above each lane. Western blot membranes were probed with human αS antibody Syn 211 (b, g), pSer129 antibodies 81A (d, h), EP1536Y (e, i) and MJF-R13 (8–8)(f, j), or anti-NFL antibody NR4 (c) as indicated above each blot. The protein bands corresponding to αS and NFL are indicated. The accumulation of aggregated, phosphorylated Ser129 αS is demonstrated in SDS-urea fraction from the brain stem/spinal cord of motor impaired M83+/+ αS (M83-I) mice. Non-αS protein bands cross-reacting with antibody pSer129/MJF-R13 (8–8) in the cortex are indicated by asterisks. The mobilities of molecular mass markers are shown on the right [file 401_2015_1485_MOESM2_ESM.pdf]

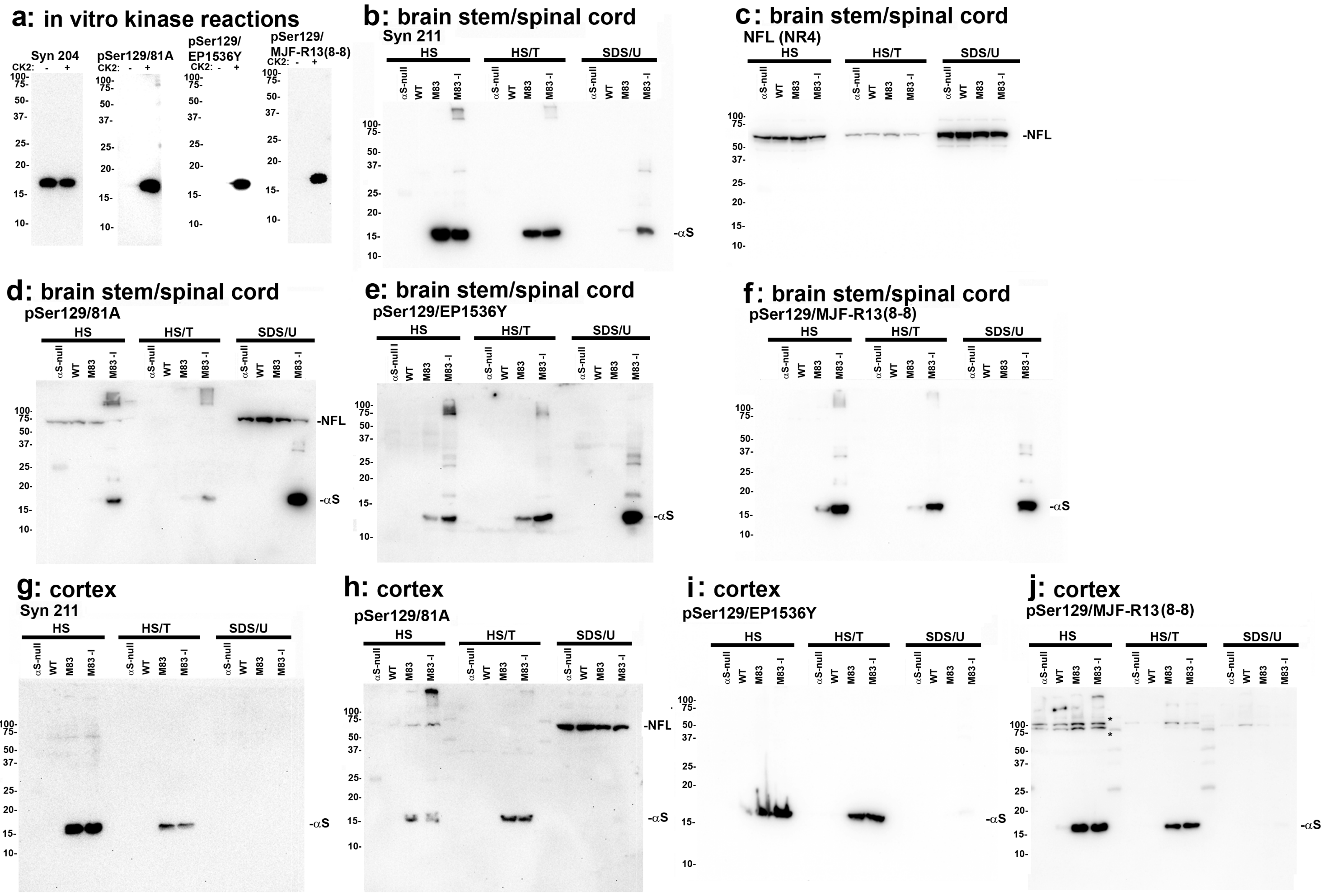

a: in vitro kinase reactions
Syn 204
pSer129/81A
pSer129/EP1536Y
pSer129/MJF-R13(8-8)
CK2: - +
b: brain stem/spinal cord
Syn 211
c: brain stem/spinal cord
NFL (NR4)
d: brain stem/spinal cord
pSer129/81A
e: brain stem/spinal cord
pSer129/EP1536Y
f: brain stem/spinal cord
pSer129/MJF-R13(8-8)
g: cortex
Syn 211
h: cortex
pSer129/81A
i: cortex
pSer129/EP1536Y
j: cortex
pSer129/MJF-R13(8-8)
HS
HS/T
SDS/U
αS-null
WT
M83
M83-I
-NFL
-αS
100-
75-
50-
37-
25-
20-
15-
10-
